# Synergistic effect of HDAC inhibitor Chidamide with Cladribine on cell cycle arrest and apoptosis by targeting HDAC2/c-Myc/RCC1 axis in acute myeloid leukemia

**DOI:** 10.1186/s40164-023-00383-5

**Published:** 2023-02-27

**Authors:** Siyu Gu, Yue Hou, Katarina Dovat, Sinisa Dovat, Chunhua Song, Zheng Ge

**Affiliations:** 1 Department of Hematology, Zhongda Hospital, School of Medicine, Southeast University, Institute of Hematology Southeast University, 87 Dingjiaqiao Street, Nanjing, 210009 China; 2 Hershey Medical Center, Pennsylvania State University Medical College, Hershey, 17033 USA; 3 Division of Hematology, The Ohio State University Wexner Medical Center, The James Cancer Hospital, Columbus, OH 43210 USA

**Keywords:** AML, HDACi, Cladribine, c-Myc, RCC1

## Abstract

**Background:**

More effective targeted therapy and new combination regimens are needed for Acute myeloid leukemia (AML), owing to the unsatisfactory long-term prognosis of the disease. Here, we investigated the synergistic effect and the mechanism of a histone deacetylase inhibitor, Chidamide in combination with Cladribine, a purine nucleoside antimetabolite analog in the disease.

**Methods:**

Cell counting kit-8 assays and Chou-Talalay’s combination index were used to examine the synergistic effect of Chidamide and Cladribine on AML cell lines (U937, THP-1, and MV4-11) and primary AML cells. PI and Annexin-V/PI assays were used to detect the cell cycle effect and apoptosis effect, respectively. Global transcriptome analysis, RT-qPCR, c-MYC Knockdown, western blotting, co-immunoprecipitation, and chromatin immunoprecipitation assays were employed to explore the molecule mechanisms.

**Results:**

The combination of Chidamide with Cladribine showed a significant increase in cell proliferation arrest, the G0/G1 phase arrest, and apoptosis compared to the single drug control in AML cell lines along with upregulated p21^Waf1/Cip1^ expression and downregulated CDK2/Cyclin E2 complex, and elevated cleaved caspase-9, caspase-3, and PARP. The combination significantly suppresses the c-MYC expression in AML cells, and c-MYC knockdown significantly increased the sensitivity of U937 cells to the combination compared to single drug control. Moreover, we observed HDAC2 interacts with c-Myc in AML cells, and we further identified that c-Myc binds to the promoter region of RCC1 that also could be suppressed by the combination through c-Myc-dependent. Consistently, a positive correlation of RCC1 with c-MYC was observed in the AML patient cohort. Also, RCC1 and HDAC2 high expression are associated with poor survival in AML patients. Finally, we also observed the combination significantly suppresses cell growth and induces the apoptosis of primary cells in AML patients with AML1-ETO fusion, c-KIT mutation, MLL-AF6 fusion, FLT3-ITD mutation, and in a CMML-BP patient with complex karyotype.

**Conclusions:**

Our results demonstrated the synergistic effect of Chidamide with Cladribine on cell growth arrest, cell cycle arrest, and apoptosis in AML and primary cells with genetic defects by targeting HDAC2/c-Myc/RCC1 signaling in AML. Our data provide experimental evidence for the undergoing clinical trial (Clinical Trial ID: NCT05330364) of Chidamide plus Cladribine as a new potential regimen in AML.

**Supplementary Information:**

The online version contains supplementary material available at 10.1186/s40164-023-00383-5.

## Background

Acute myeloid leukemia (AML) is a group of heterogeneous malignant hematological diseases with the increased capability of precursor cells’ self-renewal, proliferation and decreased capability of differentiation. The standard induction therapy, daunorubicin for 3 days and cytarabine (Ara-C) for 7 days (3 + 7 regimen), has been used over the past 50 years. However, more than 50% of patients who achieved a complete remission (CR) will eventually relapse [1]. Therefore, novel targeted drugs and new combination therapies for AML have been constantly springing up [2].

Histone deacetylase inhibitor (HDACi) is a group of epigenetic modulators, including HDAC isoform-selective inhibitors (e.g. Entinostat, Romidepsin, Trapoxin A, and Valproic acid) and against all types of HDACs (pan-inhibitors, e.g. Trichostatin A, Vorinostat, Panobinostat, Belinostat, and Pracinostat) that have been proven to have anti-leukemia effects in AML [3]. Chidamide (CHI) is a novel subtype-selective HDACi, that could selectively inhibit the activity of class I (HDAC1, 2, and 3) and class IIb (HDAC10) HDAC with a low concentration, which has been approved to treat peripheral T-cell lymphoma [4]. It has been reported that CHI could inhibit cell proliferation, induces G0/G1 arrest, increases apoptosis [5, 6], and the chemosensitivity [7, 8] alone or in combination with other drugs in AML.

Cladribine (2-chloro-2ʹ-deoxyadenosine, CLA) is a purine nucleoside antimetabolite analog that is metabolized by the purine nucleoside salvage pathways. CLA triphosphate, a phosphorylated metabolite of CLA, incorporates into DNA, resulting in single-strand breaks in DNA, and triggers apoptosis, is developed as a treatment for hematological malignancies [9, 10]. CLA shows the effective in AML and particularly when combined with Ara-C, which can modulate the bioactivation of Ara-C [11].

In the present study, we observed the synergistic anti-leukemia effect of CHI combined with CLA by inducing cell growth arrest, cell cycle arrest, and apoptosis in AML cells and identified the underlying mechanisms of the synergy through targeting HDAC2/c-Myc/RCC1 signaling in AML.

## Methods

### Clinical samples and AML primary cells

A Total of 77 bone marrow samples from AML patients and 72 control samples from healthy volunteers were collected from October 2019 to December 2021. A total of 5 AML patients presenting with high leukocyte counts consented to undergo leukapheresis to collect primary cells. Peripheral blood mononuclear cells (PBMC) from 2 healthy donors were extracted to detect the toxicity of drugs. The mononuclear cells were separated using a lymphocytes separation medium (MP biomedicals, Cat. No.S5019, USA) and erythrocytes were lysed with red blood cell lysis buffer (Biosharp, Cat. No. BL503A, China). Total RNA was isolated from samples using Trizol as described previously [12], and the complementary DNA was prepared for the detection of the target genes by Real-time quantitative Polymerase Chain Reaction (RT-qPCR). The primary cells were cultured in Roswell Park Memorial Institute 1640 medium (RPMI 1640, Gibco, USA) with 10% fetal bovine serum (FBS, Gibco, USA) and subsequently used in various experiments. The characterization of the patients is shown in Additional file 1: Table S1. The cases of AML at Zhongda Hospital Southeast University were used in this study after the acquisition of written informed consent, and by the tenets of the Declaration of Helsinki and approved by the Independent Ethics Committee for Clinical Research of Zhongda Hospital Southeast University.

### Cell lines and regents

AML cell lines U937, THP-1, and MV4-11 were purchased from the National Collection of Authenticated Cell Cultures (Shanghai, China). U937 and THP-1 cells were cultured in RPMI 1640. MV4-11 cells were cultured in Iscove’s Modified Dulbecco’s Media (IMDM, Gbico, USA). All cell culture media contained 10% FBS, and were cultured at 37 °C in a humidified atmosphere with 5% CO2.

CHI (Cat. No. S8567), and CLA (Cat. No. S1199) were purchased from Selleck. CHI and CLA powders were dissolved in dimethyl sulfoxide (DMSO, Sigma, USA) at concentrations of 10 mM, respectively for stocking.

### Bioinformatic analysis of public databases

The ChIP-seq data of hematopoietic stem and progenitor cells were used to screen c-Myc binding peaks with a threshold score > 1(Additional file 2: Table S2), downloaded from the Cistrome online tool (http://cistrome.org/db/) [13]. The RNA-sequence (RNA-seq) data and clinical data of AML patients were downloaded from The Cancer Genome Atlas (TCGA) database and Gene Expression Omnibus (GEO) database. The Spearman correlation analysis of MYC and RCC1 expression utilized the RNA profile data from TCGA-LAML, GSE995, and GSE5122. Data from GSE13159 and GSE114868 were used to compare the RNA expression between AML patients and healthy donors. The log-rank survival analysis utilized the clinical data from the TCGA-LAML, GSE12417, and GSE37642 datasets.

### Cell proliferation assay

The cell counting kit-8 (CCK-8, Dojindo, Japan) was used for cell proliferation assay as we previously described [12]. Briefly, cells were plated into a 96-well plate at a density of 1 × 10^5^ cells/mL with 100 μL of complete medium per well and cultured for 24–72 h with exposure to the indicated drugs, 10 μL of CCK-8 reagent was added to each well before 2–4 h of the experiment end. Absorbance detection was performed at 450 nm using a microplate reader (Bio-Tek, USA). The cell survival rate was calculated as follows:

$$\left[ \left( {{\text{OD450 test group}} - {\text{OD450 blank}}} \right)/\left( {{\text{OD450 control group}} - {\text{OD450 blank}}} \right)\right ]\, \times \,\,100\%$$

The 50% inhibitory concentration (IC50) was defined as the half maximal inhibitory concentration, which represents the concentration of an inhibitor that is required for 50% inhibition of cells. The synergistic effect was analyzed by CompuSyn software (ComboSyn, Inc., Paramus, NJ. 07652 USA). Through the combination index (CI) theorem from Chou-Talalay’s quantitative definition of drug combinations: additive effect (CI = 1), synergism (CI < 1), and antagonism (CI > 1) [14].

### Plasmid construction, lentiviral transduction, and c-MYC Knockdown

Lentiviral shRNA plasmids for c-MYC were constructed by subcloning the shRNA oligos into the lentiviral shRNA vector with an IRES GFP (pLV3ltr-ZsGreen-Puro-U6) (Corues Biotechnology, China) as the manufacturer's instruction. The DNA sequence of the shRNA oligos was listed in Additional file 3: Table S3. To generate the lentivirus, 293T cells were transfected with the resulting shRNA plasmid and packaged plasmids (psPAX2 and pMD2.G) (Corues Biotechnology, China), via Effect Transfection Reagent (Vazyme Biotech Co., Ltd, China, NO. T101-01). Viral Supernatant was collected and filtered with a 45 μM filter 48 h and 72 h post-transfection. U937 cells were then transduced with the resulting lentivirus. Protamine sulfate (Beyotime Biotechnology) was added to increase the transduction efficiency. 48 h post-transduction, infected cells were treated with 5 ug/mL puromycin (InvivoGen, CAS.58582) for stable expressing cell selection. The efficiency of transduction was monitored by GFP (+) cells via flow cytometry.

### Cell cycle assay

Cell cycle analysis was performed as we previously reported [12]. Briefly, cells were fixed overnight in 70% precooled ethanol at 4 °C and stained with 0.5 mL of propidium iodide (PI)/RNase Staining Buffer (BD Biosciences, Cat. No. 550825) for 15 min at room temperature. The DNA content was assessed by flow cytometry (Attune NxT Flow Cytometer, Thermo Fisher Scientific Co., Ltd., USA) at an excitation wavelength of 488 nm and an emission wavelength of 585 ± 21 nm. ModfitLT software version 5.0 (Verity Software House, Inc., Topsham, ME, USA) was used for the analysis of DNA distributions.

### Apoptosis assay

Apoptosis assay was carried out as we previously reported [12]. Briefly, cells were incubated with 5 μL of Annexin V-FITC and 5 μL of PI (BD Biosciences, Cat. No. 556547) or 5 μL of Annexin V-APC (BD Biosciences, Cat. No. 550474) and 5 μL of 7AAD (BD Biosciences, Cat. No. 559925) for 15 min in the dark. Apoptosis was detected by flow cytometry at an excitation wavelength of 488 nm and emission wavelengths of 525 ± 20 nm and 585 ± 20 nm. FlowJo version 10.0 software was used to analyze the number and percentage of apoptotic cells.

### Whole transcriptome analysis

RNA sequencing was performed for the whole transcriptome analysis. U937 cells were treated with 8 μM CHI or 0.1 μM CLA or DMSO control for 48 h. Cells were harvested and total RNA was isolated with Trizol reagent. Sequencing libraries were generated using NEBNext^®^ UltraTM RNA Library Prep Kit for Illumina^*®*^ (NEB, USA) following the manufacturer’s recommendations and index codes were added to attribute sequences to each sample. The clustering of the index-coded samples was performed on a cBot Cluster Generation System using TruSeq PE Cluster Kit v3-cBot-HS (Illumia) according to the manufacturer’s instructions. The resulting libraries were sequenced by Novogene (Beijing, China) on a HiSeq device (Illumina, San Diego, CA, USA) to yield a transcriptome. The index of the reference genome was built using Hisat2 v2.0.5 and paired-end clean reads were aligned to the reference genome using Hisat2 v2.0.5. featureCounts v1.5.0-p3 was used to count the read numbers mapped to each gene. Significant differences in expression were analyzed using DESeq2. Genes with absolute fold changes > 1 and a Benjamini–Hochberg adjusted *p* < 0.05 were validated further. The enrichment analysis utilized the online website tool Metascape (http://metascape.org/) referred to different databases.

### RT-qPCR

The total RNA was reversed and transcribed into cDNA using the PrimeScript™ RT Master Mix (Perfect Real Time) (TaKaRa, Cat. No. RR036A, China). RT-qPCR was performed using SYBR Supermix (TaKaRa, Cat. No. RR420A, China) on a StepOne Plus analysis system (Applied Biosystems, Foster City, CA, USA). The amplification conditions were as follows: pre-denaturation (95 °C for 30 sec), 40 cycles of denaturation (95 °C for 30 sec), and annealing and extension (60 °C for 30 sec). The DNA sequences of the qPCR primers for each target gene were listed in Additional file 3: Table S3. The relative expression levels of the target genes were calculated by the comparative Ct method presented as 2^−ΔΔCt^ for cell lines and 2^−ΔCt^ for clinical samples.

### Co-immunoprecipitation (co-IP)

A total of 2–5 × 10^7^ cells were washed with PBS and lysed in lysis buffer (Cell lysis buffer for Western and IP, Beyotime Biotechnology, Cat. No. P0013) containing protease inhibitor (Beyotime Biotechnology, Cat. No. P1045) for 30 min on ice. After centrifugation, the supernatant was collected and precleared with Protein A/G MagBeads (GenScript, Cat. No. L00277, USA) at 4 °C for 60 min. Protein concentrations were normalized by the BCA protein assay kit (Beyotime Biotechnology, Cat. No. P0011). An approximate 50 μL whole cell lysate was taken as input for each IP. The cell lysate was then incubated with his antibody, as well as precleaned Protein A/G MagBeads overnight at 4 °C. Immunoprecipitates were collected by centrifugation and washed three times with PBS. Then the beads complexed with the immunoprecipitated proteins were resuspended in sodium dodecyl sulfate–polyacrylamide gel electrophoresis (SDS-PAGE) sample loading buffer and boiled at 100 °C for 15 min, processed for Western blotting analysis.

### Western blot (WB)

Cells were lysed by RIPA Lysis Buffer (Beyotime Biotechnology, Cat. No. P0013B) containing Phenylmethanesulfonyl fluoride (PMSF, Beyotime Biotechnology, Cat. No. ST506). The total protein concentration was measured using a BCA Protein Assay Kit. Then, 30 µg of each protein sample was separated using an SDS-PAGE sample loading buffer (Beyotime Biotechnology, Cat. No. P0015) and transferred to a polyvinylidene difluoride membrane (Millipore Corporation, Billerica, USA). The primary antibodies were summarized in Additional file 4: Table S4. After three washes with tris-buffered saline plus Tween-20, the membranes were incubated with appropriate secondary antibodies (Jackson Immuno Research, USA). Finally, Pierce™ ECL Plus Western Blotting Substrate (Thermo Fisher Scientific, USA) was used to visualize the reactive proteins. Conventional western blot protocols were used throughout.

### Chromatin immunoprecipitation (ChIP) assays

ChIP assay was carried out as we previously reported [15]. Briefly, a total of 2 × 10^7^ U937 cells were fixed with 1% formaldehyde (Sigma Aldrich, USA, Cat. No. 252549) for 10 min on ice. After being neutralized by glycine (Beyotime Biotechnology, Cat. No. ST085), a terminal of 2 × 10^6^ cells was resuspended in a 300 μL lysis buffer with protease inhibitor (Beyotime Biotechnology, Cat. No. P1045). The cells were lysed by ultrasonication using a sonicator (Bioruptor Pico, Belgium) at 25% power, 30-sec shock, and 30-sec gap, for a total of 30 times to shear DNA to an average length ranging from 200 to 1000 bp. Then chromatin was immunoprecipitated with 5 μg anti-c-Myc antibody, and 1 μg of Normal IgG (Additional file 4: Table S4) as the negative control. The promoter region defaulted the sequence from − 2000 to + 100 bp of RCC1. Final DNA extractions were amplified with qPCR using primer pairs that cover the potential c-Myc binding site that was predicted using JASPAR online tool (https://jaspar.genereg.net/) [16]. The DNA sequence of the primers was listed in Additional file 3: Table S3.

### Statistical analysis

All data were analyzed with the IBM SPSS statistics 26 (IBM Corp., Armonk, NY, USA) and GraphPad Prism 8 (Graph Pad Inc., San Diego, CA, USA) software, and the results were expressed as the mean of three independent experiments ± standard deviation (SD) the mean, analyzed by variance (ANOVA) test for multiple groups and by two-tailed t-test for double groups. Clinical samples data was performed with the Mann–Whitney U test for continuous variables and Fisher's exact test for categorical variables. Spearman correlation analysis was used for the correlation study of genes, and survival curves were performed using a Log-rank (Mantel-Cox) test. *p* < 0.05 was considered to be statistical significance.

## Results

### Chidamide inhibits the proliferation of acute myeloid leukemia cells

**Fig. 1:**
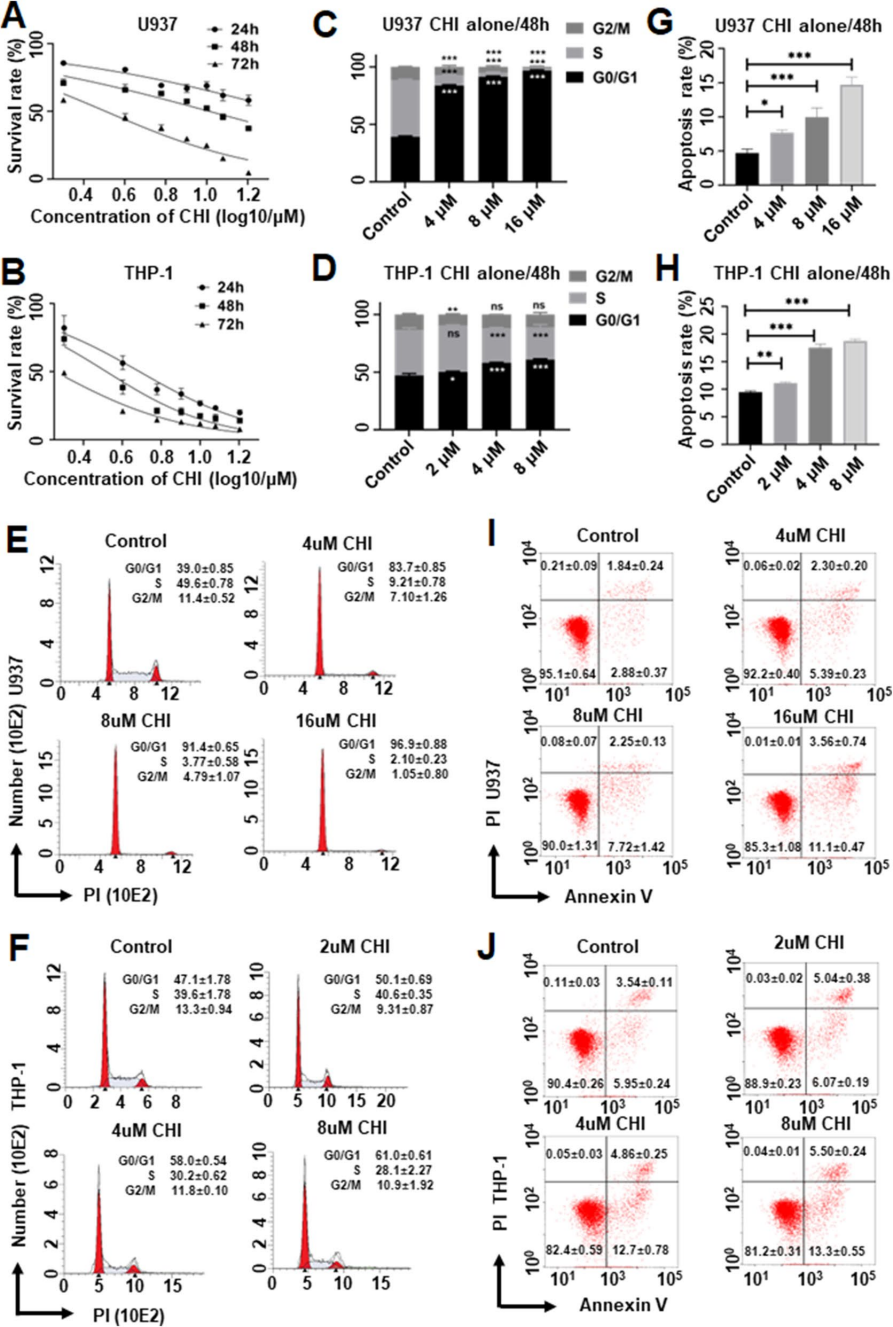
The inhibition effects of Chidamide (CHI) alone in AML cells. The cell proliferation assays in (**A**) U937 cells and (**B**) THP-1 cells were treated by CHI alone with gradient concentration for 24, 48, and 72 h. The statistic histogram and flow cytometry histogram of cell cycle effect in (**C**, **E**) U937 cells and (**D**, **F**) THP-1 cells that were treated by CHI alone with gradient concentration for 48 h. The statistic histogram and flow cytometry scatter plot of apoptosis effect in (**G**, **I**) U937 cells and (**H**, **J**) THP-1 cells that were treated by CHI alone with gradient concentration for 48 h. *p < 0.05, **p < 0.01, ***p < 0.001

To explore the effect of CHI on cell proliferation, AML cell lines, U937, THP-1, and MV4-11 were treated with different doses of CHI for 24, 48, and 72 h, and the IC50 was achieved for each cell line (Additional file 5: Table S5). Results showed that CHI inhibited cell viability in a dose- and time-dependent manner (Fig. 1A, B, Additional file 6: Fig. S1A). Cell cycle analysis was also analyzed in the cells treated with half IC50, IC50, and double IC50 for 48 h, and the G0/G1 phase was significantly increased upon CHI treatment in a dose-dependent manner. The percentage of G0/G1 phase was significantly increased in U937 cell by CHI compared with control (control: 39.04 ± 0.85% vs. CHI 4 µM: 83.79 ± 0.85%, *p* < 0.001; vs. CHI 8 µM: 91.44 ± 0.65%, *p* < 0.001; vs. CHI 16 µM: 96.85 ± 0.88%, *p* < 0.001) (Fig. 1C, E). Similar results were also observed in THP-1 (Fig. 1D, F) and MV4-11 (Additional file 6: Fig. S1B, C) cells. Moreover, apoptosis was also examined in the cells treated with the same doses of CHI for 48 h. The results showed that CHI treatment significantly increased apoptosis rate versus control in U937 cells (Fig. 1G, I), THP-1 (Fig. 1H, J), and MV4-11 (Additional file 6: Fig. S1D, E) cells in a dose-dependent manner.

### The synergistically effect of Chidamide plus Cladribine in AML cells

**Fig. 2:**
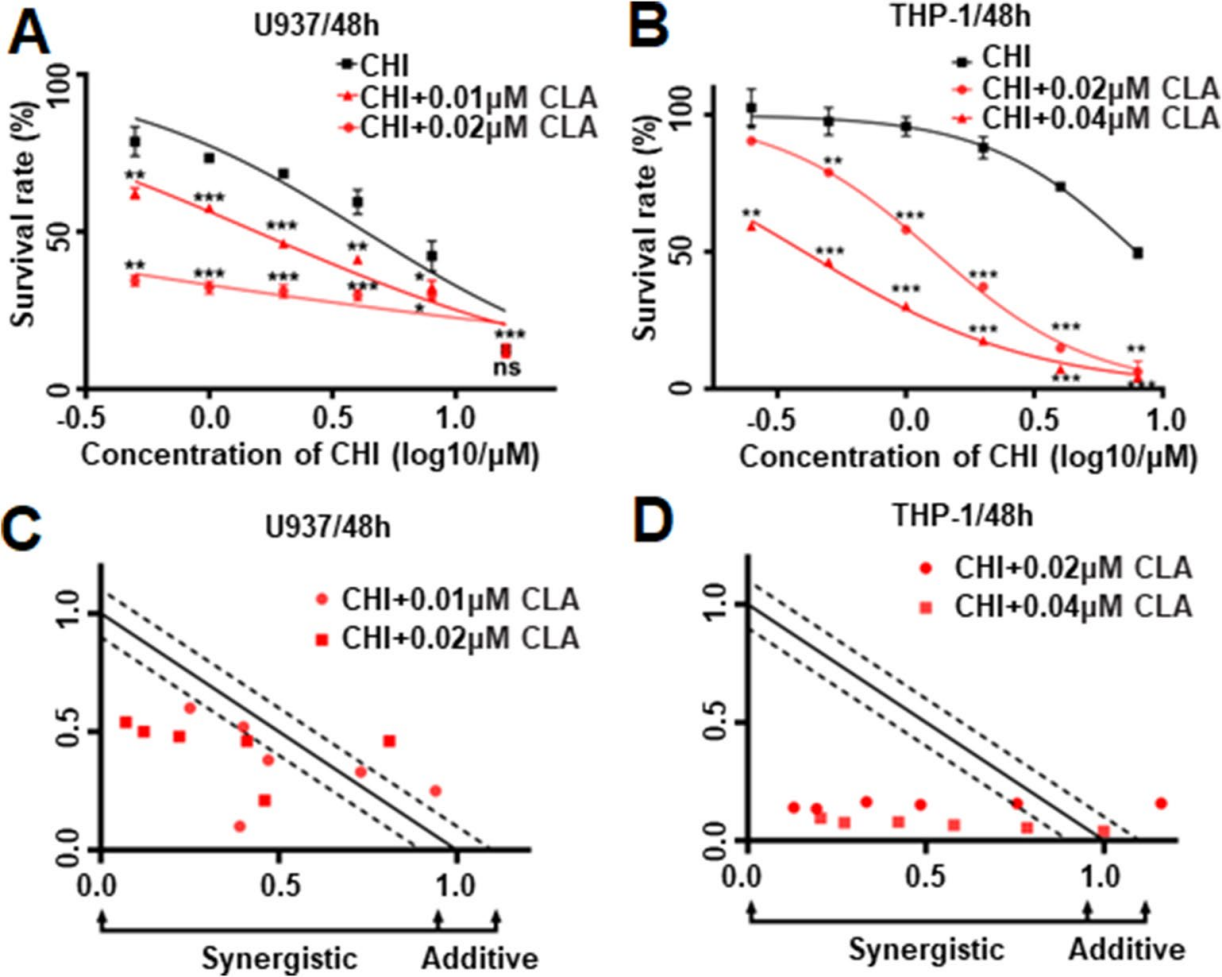
The synergistic effect of Chidamide (CHI) and Cladribine (CLA) in AML cells. The cell proliferation assays in (**A**) U937 cells and (**B**) THP-1 cells were treated by CHI alone with gradient concentration or CHI combined with 0.01, 0.02 or 0.04 μM CLA for 48 h. The normalized isobologram plot of the combination of CHI and CLA in (**C**) U937 cells and (**D**) THP-1 cells. *p < 0.05, **p < 0.01, ***p < 0.001

Both CHI and CLA have currently used as clinical drugs. We first explored the cytotoxic effect of the combination of CHI with CLA on PBMC from healthy donors, and results showed there is no significantly enhanced effect of CHI with CLA on the PBMC compared to that of the single drug controls (Additional file 7: Fig. S2A–D). The effect of CLA on cell proliferation arrest in AML cells and calculated the IC50s (Additional file 7: Fig. S2E–G). Then, we observed the cell proliferation rate in the AML cells treated with various doses of CHI in the combination of IC50 or half IC50 CLA for 48 h. Results showed that cell proliferation is significantly inhibited in the combination of CHI with CLA compared to either a single drug alone or DMSO control in U937, THP-1, and MV4-11 cells (Fig. 2A, B, Additional file 7: S2H). CompuSyn analysis showed the combination had a synergistic effect on cell proliferation arrest in the three cell lines (Fig. 2C, D, Additional files 7, 8: Fig. S2I, Table S6).

### Chidamide plus Cladribine induced cell cycle arrest effect in AML cell lines

**Fig. 3:**
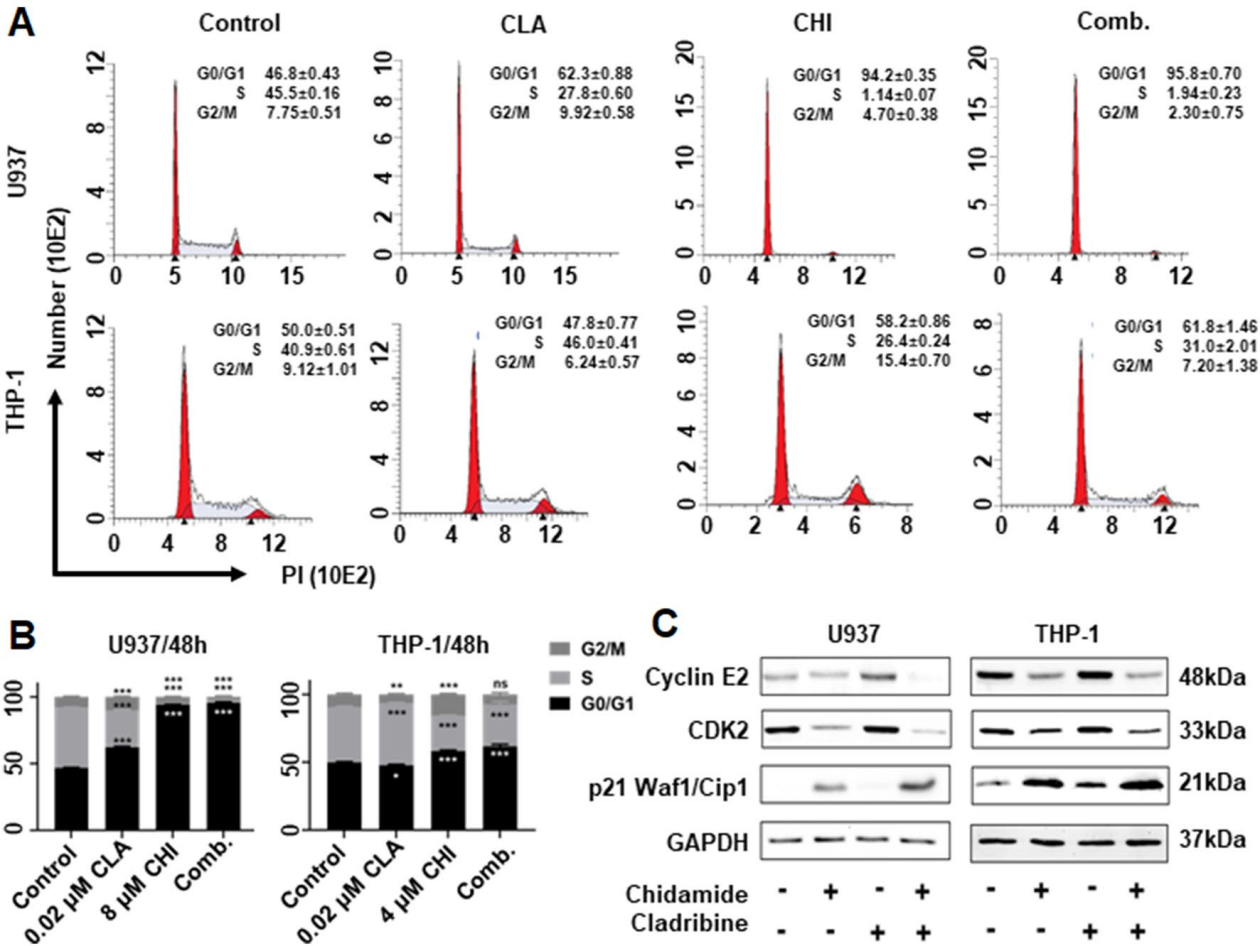
The synergistic cell cycle effect of Chidamide (CHI) and Cladribine (CLA) in AML cells. The flow cytometry histogram (**A**) and statistic histogram (**B**) of cell cycle effect in U937 cells and THP-1 cells that were treated by CHI alone, CLA alone, and two drugs combination for 48 h. (**C**) Western blot of cyclin E2, CDK2, and p21 ^Waf1/Cip1^ in U937 cells and THP-1 cells that were treated with CHI alone, CLA alone, and two drugs combination for 48 h. *p < 0.05, **p < 0.01, ***p < 0.001

We next explored the effect of CHI with CLA on cell cycle arrest in AML cells. Results showed that the combination of CHI with CLA significantly increased the G0/G1 phase arrest in U937, THP-1, and MV4-11 compared to single drug control (Fig. 3A, Additional file 9: Fig. S3A). Quantitation data showed that CLA significantly induced the G0/G1 phase arrest at the low dose (0.02 µM) compared to DMSO control in U937 (control: 46.79 ± 0.43% vs. 62.27 ± 0.88%, *p* < 0.001), MV4-11 (60.20 ± 1.39% vs. 63.17 ± 0.24%, *p* < 0.001), but not in THP-1 cell line (49.95 ± 0.52% vs. 47.75 ± 0.77%, *p* > 0.05) (Fig. 3B, Additional file 9: Fig. S3B). We also explore the effect of the combination on the expression of the genes critical for the G0/G1 phase progress by WB. Results showed that the combination treatment significantly increased levels of p21 ^Waf1/Cip1^ proteins, a key cyclin-dependent kinase inhibitor family member, but decreased the CDK2 and Cyclin E2, the rate-limiting pathway(s) factor for the progress of G1 to S cell phase, compared to the single drug control (Fig. 3C, Additional file 9: Fig. S3C).

### Chidamide plus Cladribine induced apoptosis effect in AML cells lines

**Fig. 4:**
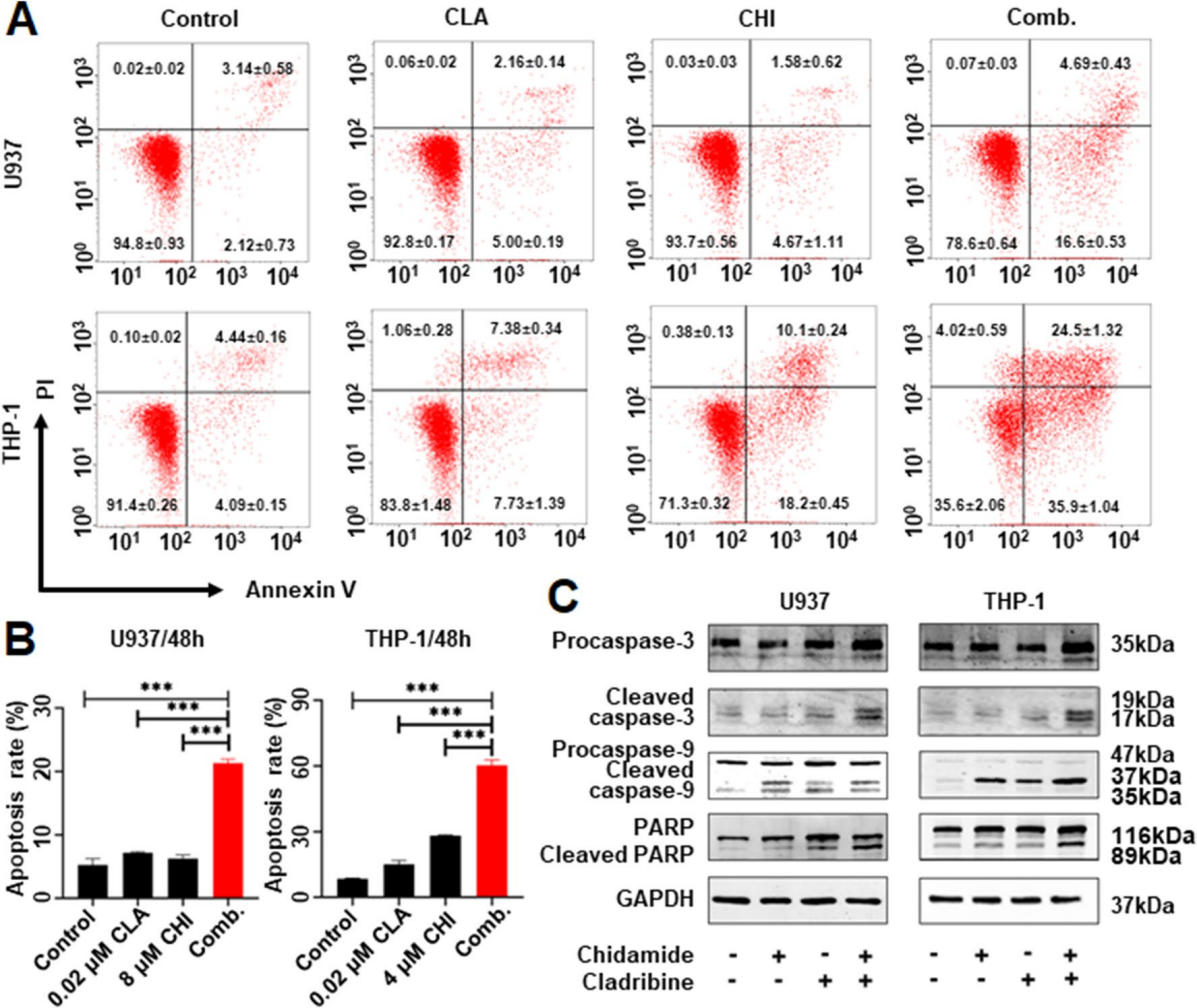
The synergistic apoptosis effect of Chidamide (CHI) and Cladribine (CLA) in AML cells. The flow cytometry scatters plot (**A**) and statistic histogram (**B**) of apoptosis effect in U937 cells and THP-1 cells that were treated by CHI alone, CLA alone, and two drugs combination for 48 h. (**C**) Western blot of caspase-9 and the cleaved one, caspase-3 and the cleaved one, PARP and the cleaved one in U937 cells and THP-1 cells that were treated with CHI alone, CLA alone, and two drugs combination for 48 h. *p < 0.05, **p < 0.01, ***p < 0.001

We also explored the effect of CHI with CLA on apoptosis in AML cells. Results showed that the combination of CHI with CLA significantly increased the apoptosis compared to CHI single drug control in THP-1 (60.37 ± 2.36% vs. 28.24 ± 0.39%, *p* < 0.01), U937 (21.29 ± 0.66% vs. 6.25 ± 0.56%, *p* < 0.001) and MV4-11(17.43 ± 0.26% vs. 9.89 ± 1.62%, *p* < 0.001) and also compared to the low dose of CLA alone (Fig. 4A, B, Additional file 10: Fig. S4A, B). Consistently, WB data showed that the combination significantly increased the expression of the apoptosis-related proteins, cleaved caspase-3, cleaved poly (ADP-ribose) polymerase (PARP), and cleaved caspase-9 compared to the single drug control (Fig. 4C, Additional file 10: S4C).

### The key molecule c-Myc in the anti-leukemia effect of the combination

**Fig. 5:**
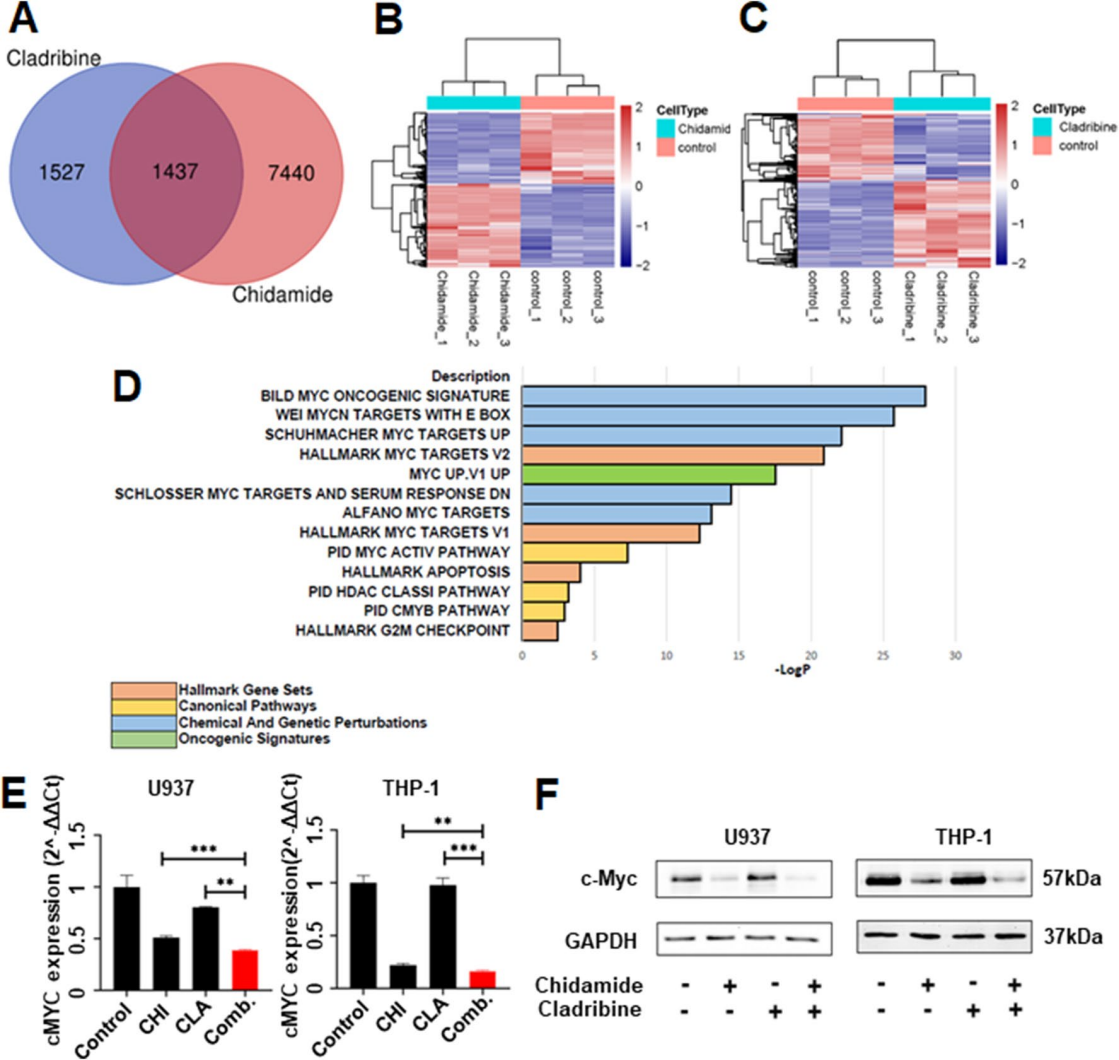
The RNA-seq analysis of Chidamide (CHI) and Cladribine (CLA) in U937 cells and the change of c-Myc with different treatments. (**A**) The Venn plot shows the overlapped differential expression genes (DEGs) of U937 cells that were treated by CHI and CLA. The heat maps of the co-DEGs in U937 cells were treated with CHI (**B**) and CLA (**C**). (**D**) The enrichment analysis of the co-DEGs. (**E**) The expression of c-MYC in U937 cells and THP-1 cells treated with CHI alone, CLA alone, and two drugs combination for 48 h was detected by RT-qPCR. (**F**) Western blot of c-Myc in U937 cells and THP-1 cells that were treated by CHI alone, CLA alone, and two drugs combination for 48 h. *p < 0.05, **p < 0.01, ***p < 0.001

To understand the molecular mechanisms underlying the synergy of CHI with CLA on cell cycle arrest and apoptosis, we performed the global transcriptome analysis in U937 cells treated with CHI or CLA, respectively. A total of 2964 and 8877 differential expression genes (DEGs) were identified upon the treatment of CHI and CLA, respectively compared to the DMSO control, in which 1437 DEGs were overlapped by a Venn diagram plot (Fig. 5A). The heatmap clearly showed the difference of the overlapped DEGs upon the drug treatment versus DMSO control (Fig. 5B, C). We identified that MYC oncogenic signaling was the top pathway upon the drug treatment (Fig. 5D) by different computer tool analyses, Hallmark Gene Sets (Additional file 11: Fig. S5A), Canonical Pathways (Additional file 11: Fig. S5B), Chemical and Genetic Perturbations (Additional file 11: Fig S5C), and Oncogenic Signatures datasets (Additional file 11: Fig. S5D). Indeed, RT-qPCR data and WB indicated the CHI significantly down-regulated the mRNA and protein level of c-MYC in U937, THP-1, and MV-4-11 cells compared to the DMSO control, and the combination of CHI with CLA further decreased the c-Myc level compared to single drug control (Fig. 5E, F, Additional file 11: Fig. S5E, F).

**Fig. 6:**
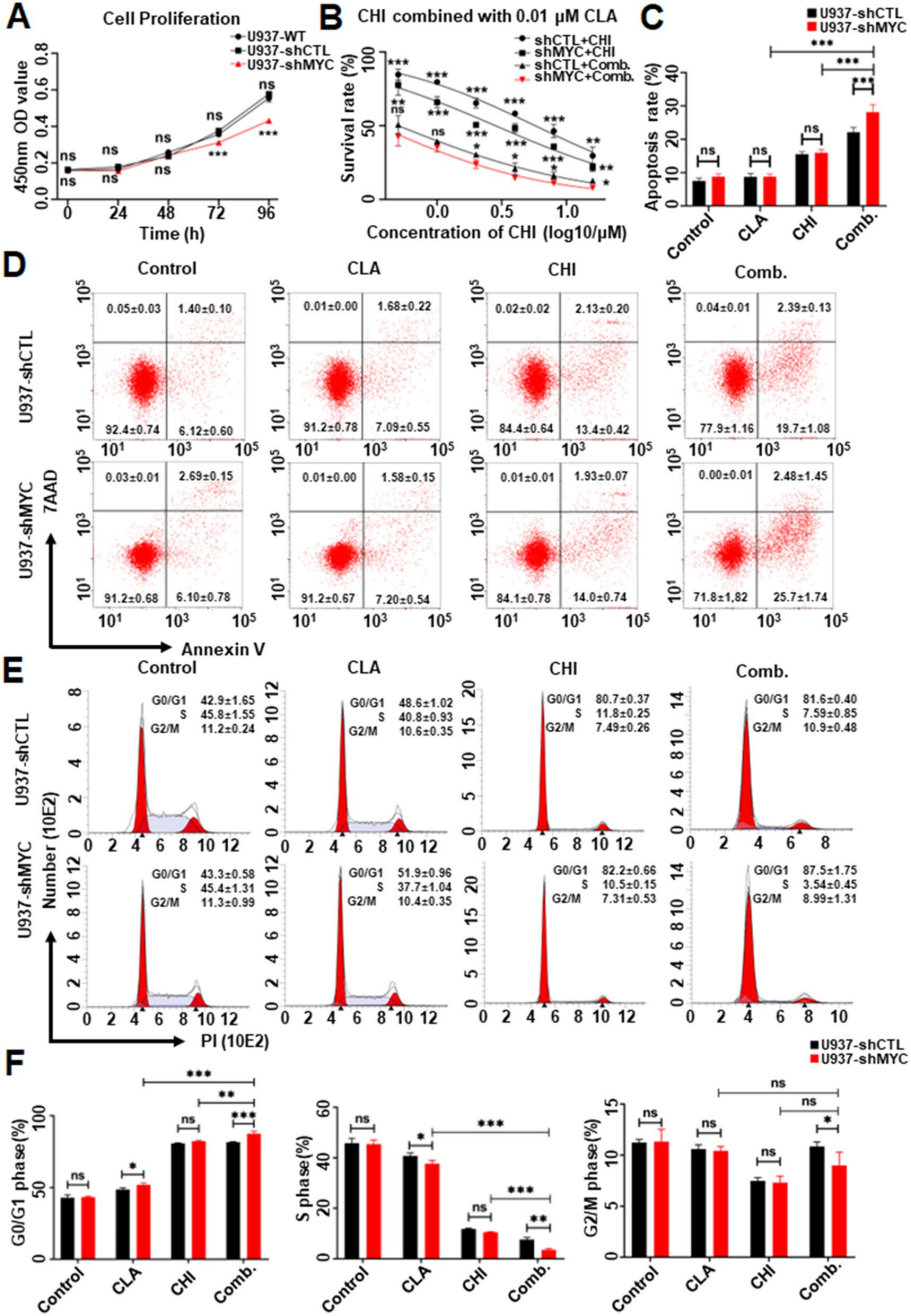
The synergistic effect of Chidamide (CHI) and Cladribine (CLA) in c-MYC knockdown U937 cells versus the scramble shRNA control (shCTL) cells. (**A**) Cell viability effect of wide type (WT), shCTL, and shMYC-U937 cells. (**B**) The inhibition effect of CHI plus 0.01 μM CLA in shCTL, and shMYC-U937 cells. (**C**) The statistic histogram and (**D**) the flow cytometry scatters plot of apoptosis effect in shCTL versus shMYC-U937 cells that were treated by CHI alone, CLA alone, and two drugs combination for 48 h. (**E**) The flow cytometry histogram and (**F**) statistic histogram of cell cycle effect in shCTL versus shMYC-U937 cells that were treated by CHI alone, CLA alone, and two drugs combination for 48 h. *p < 0.05, **p < 0.01, ***p < 0.001

We detected higher c-Myc levels in U937 and MV4-11 cell lines than that in THP-1 cell lines (Additional file 11: Fig. S5G). Then, we observed the effect of c-MYC knockdown on cell proliferation arrest in U937 cells. Results showed that c-MYC knockdown significantly inhibited the proliferation of U937 cells compared to the scramble shRNA control (shCTL) (Fig. 6A). Also, c-MYC knockdown significantly increased the sensitivity of cells to the combination of CHI with CLA on cell proliferation arrest at 48 h compared to CHI alone (Fig. 6B). Similarly, c-MYC knockdown significantly elevated the combination-induced apoptosis (Fig. 6C, D) and G0/G1 arrest (Fig. 6E, F) in the cells compared to shMYC only and single drug control.

### HDAC2 interacts with c-Myc and plays as a prognostic factor in AML patients

**Fig. 7:**
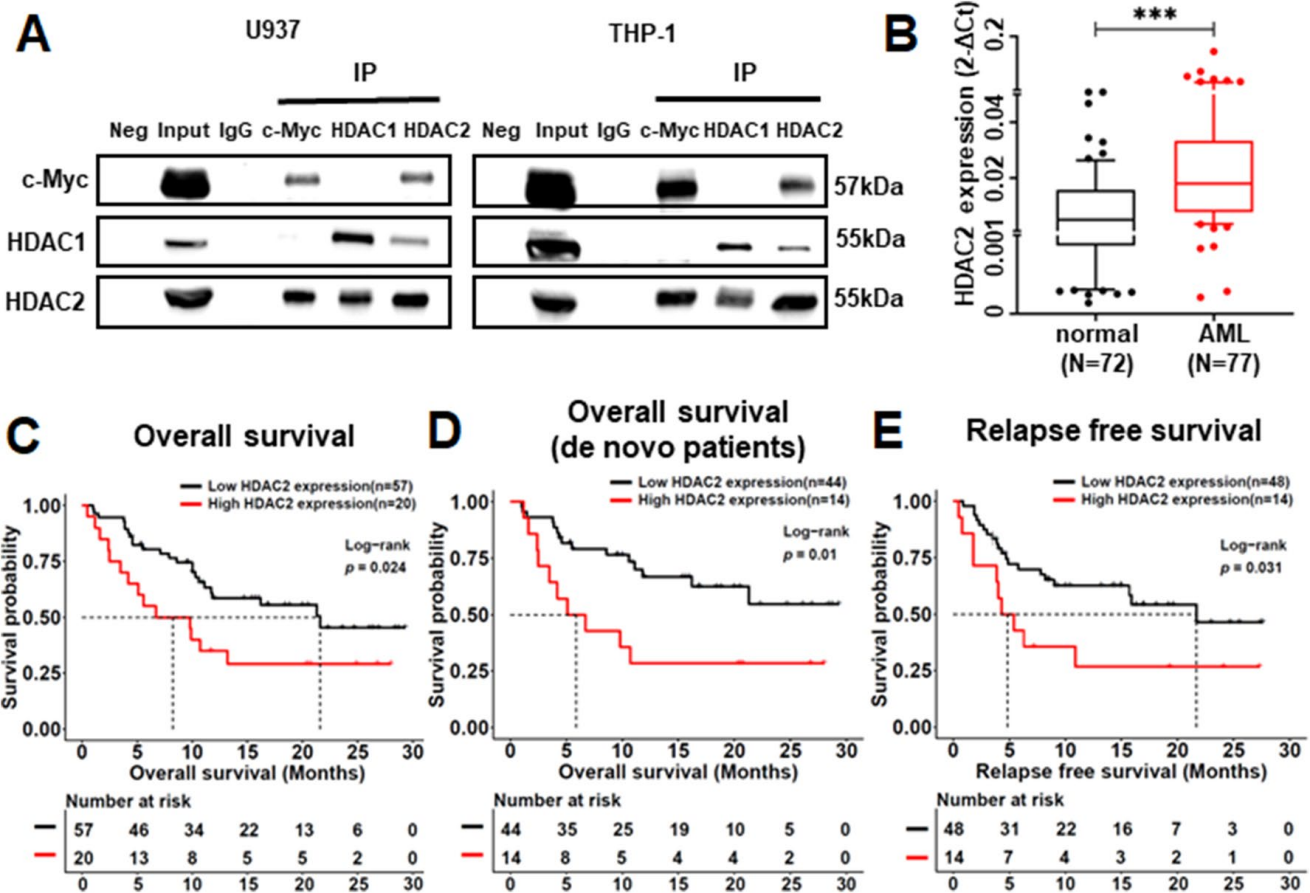
HDAC2 plays as an interactor with c-Myc and an indicator for AML prognosis. (**A**) Co-Immunoprecipitation analysis for c-Myc, HDAC1, and HDAC2 in U937 and THP-1 cells. (**B**) The elevated expression levels of HDAC2 in AML versus normal donors. The overall survival curves of HDAC2^high^ or HDAC2^low^ groups in AML patients cohort (**C**) and de novo cohort (**D**). The relapse-free survival curves of HDAC2^high^ or HDAC2^low^ groups in AML patients cohort (**E**). *p < 0.05, **p < 0.01, ***p < 0.001

Since CHI alone could significantly down-regulated c-Myc, we speculated HDACs may be directly associated with c-Myc as reported in solid tumors [17, 18], and performed a co-IP assay in AML cells. Results showed that IP with c-Myc could pull down the HDAC2 not the HDAC1, reversely, HDAC2 not HDAC1 could immunoprecipitate c-Myc in U937, THP-1, and MV-11 cells (Fig. 7A, Additional file 12: Fig. S6A). We also detected the different expression levels of HDAC2 in three AML cell lines (Additional file 12: Fig. S6B). These data indicated that HDAC2 interacts with c-Myc, which may be responsible for the combination-induced down-regulation of c-Myc in AML cells.

To determine the critical role of HDAC2 in AML, we detected the HDAC2 expression in 77 AML patients and 72 healthy donors by RT-qPCR and found that HDAC2 was highly expressed in patients (Fig. 7B). The same results were also found in GSE13159 (Additional file 12: Fig. S6C) and GSE114868 (Additional file 12: Fig. S6D) datasets but were not found in the HDAC1, HDAC3, and HDAC10. Then, we divided the AML patients into the HDAC2^high^ (top 25%, n = 20) and the HDAC2^low^ (bottom 75%, n = 57) expression groups based on the mRNA intensity. The proportion of − 5/5q in patients was much higher in the HDAC2^high^ than that in the HDAC2^low^ group [20% vs. 3.5%, *p* < 0.05]. There was no statistically different in other clinical characteristics or genetic abnormalities of the patients in the two groups (Additional file 13: Table S7). The survival curves show that patients with high HDAC2 were significantly associated with shorter overall survival (OS, all patients cohort, Fig. 7C, *p* < 0.05; de novo cohort, Fig. 7D, *p* < 0.05), and relapse-free survival (RFS, all patients cohort, Fig. 7E, *p* < 0.05) compared to that with low HDAC2. The same OS results were detected in AML patients from GSE12417-GPL570, and GSE37642-GPL570 datasets (Additional file 12: Fig S6E, F). These results indicate that HDAC2 was highly expressed in AML patients and HDAC2 high expression is related to poor outcomes.

### Association of RCC1 as the downstream effector of c-Myc with clinical features in AML patients

**Fig. 8:**
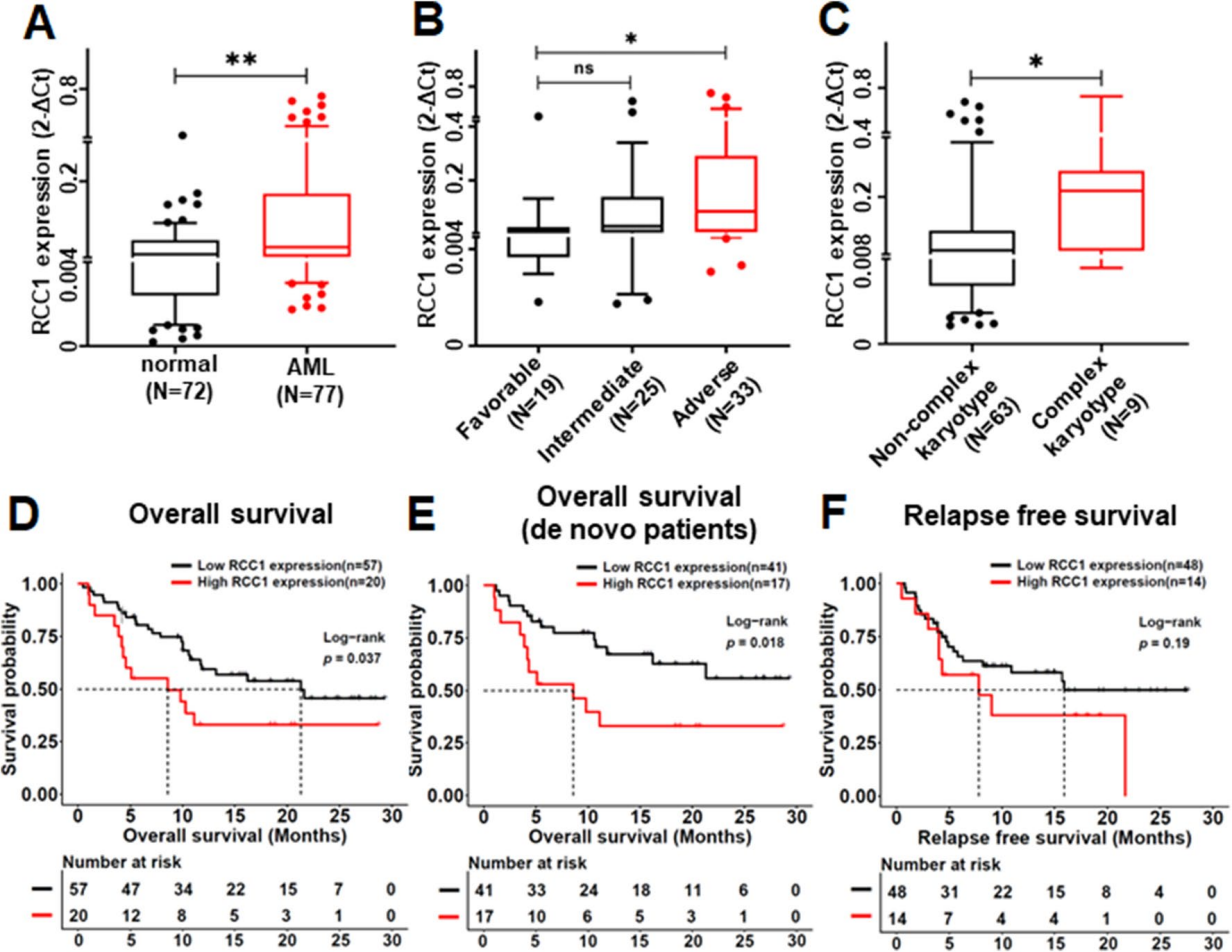
The elevated expression levels of RCC1 in AML patients’ bone marrow samples in our clinical center analyzed by RT-qPCR. (**A**) The elevated expression levels of RCC1 in AML versus normal donors. (**B**) The elevated expression levels of RCC1 in AML patients from the Adverse group and Intermediate group compare to the Favorable one. (**C**) The elevated expression levels of RCC1 in AML patients with complex karyotypes. The overall survival curves of RCC1^high^ or RCC1^low^ groups in AML patients cohort (**D**) and de novo cohort (**E**). The relapse-free survival curves of RCC1^high^or RCC1^low^ groups in AML patients cohort (**F**). *p < 0.05, **p < 0.01, ***p < 0.001

To identify the downstream target of c-Myc, we then analyzed the ChIP-seq data of hematopoietic stem and progenitor cells with the Cistrome online tool. A total of 2848 genes with c-Myc binding peaks were identified. Then, we further identified 154 candidate c-Myc target genes by overlapping the 1437 DEGs regulated by two drugs and the 2848 potential c-Myc binding genes (Additional file 14: Fig. S7A). In the c-Myc target genes, we found that RCC1 was positively correlated with c-MYC in AML patient RNA-seq cohorts from TCGA and GEO databases (GSE995 and GSE5122) (Additional file 14: Fig. S7B–D). This droves us to further explore the clinical significance of RCC1 expression by RT-qPCR, and we found that RCC1 was highly expressed in AML patients (Fig. 8A). The same results were also found in GSE13159 (Additional file 14: Fig. S7E) and GSE114868 (Additional file 14: Fig. S7F) datasets. Then, we divided the AML patients into the RCC1^high^ (top 25%, n = 20) and the RCC1^low^ (bottom 75%, n = 57) expression groups based on the mRNA intensity. The clinical characteristics of the patients in the two groups were summarized in Table 1. The BM blasts at diagnosis were much higher in the RCC1^high^ than that in the RCC1^low^ group [72.4 (12.4–96.0) vs. 43 (6.05–94.8), *p* < 0.05]. A significantly lower CR rate after induction therapy was observed in RCC1^high^ versus those in the RCC1^low^ group (55.0% vs. 84.2%, *p* < 0.05). According to 2017 ELA risk stratification [19], the proportion of adverse cohort was significantly elevated in RCC1^high^ than that in the RCC1^low^ group (70.0% vs. 33.3%, *p* < 0.05), and the expression of RCC1 were also much higher in the adverse cohort compared to the favorable one (*p* < 0.05, Fig. 8B). There was not statistically different in gender, age, WBC, Hb, PLT and FAB subtypes between the RCC1^high^ and RCC1^low^ groups in our cohort. In Table 2, we summarized the genetic abnormalities of patients and found significantly higher rates of KMT2A rearrangement (20.0% vs. 3.5%, *p* < 0.05) and complex karyotype (37.5% vs. 5.4%, *p* < 0.05) in RCC1^high^ group and the RCC1 expression was also significantly elevated in the patients with complex karyotypes (Fig. 8C, *p* < 0.05). The survival curves show that in all patients cohort (Fig. 8D, *p* < 0.05) and the de novo cohort (Fig. 8E, *p* < 0.05), patients with high RCC1 were significantly associated with shorter OS compared to that with low RCC1. A similar but nonsignificant trend was also found for RFS (Fig. 8F, *p* = 0.19). The same OS results were detected in AML patients from the TCGA-LAML, GSE12417-GPL570, and GSE37642-GPL570 datasets (Additional file 14: Fig S7G–I). Taken together, these results showed that the RCC1 expression was elevated in AML patients and RCC1-high expression was associated with poor outcomes in AML patients.

**Table 1:** Clinical characteristics of AML patients respect to RCC1 expression

Characteristics	total (n = 77)	low RCC1 expression (n = 57)	high RCC1 expression (n = 20)	*p*-value
	n (%) or median (range)	n (%) or median (range)	n (%) or median (range)	
Gender (Male)	40 (51.9%)	29 (550.9%)	11 (55.0%)	0.799
Age (years)	60 (18–86)	60 (18–81)	67.5 (24–86)	0.093
WBC (× 10^9^/L)	7.71 (0.4–123.6)	7.79 (0.4–123.6)	6.0 (0.69–100.4)	0.830
Hb (g/L)	75 (36–152)	75 (36–124)	77 (42–152)	0.693
Plt (× 10^9^/L)	53 (3–259)	50 (3–259)	53.3 (15–140)	0.542
BM blast (%)	46 (6.05–96)	43 (6.05–94.8)	72.4 (12.4–96)	**0.027**
FAB				
M1	7 (9.09%)	3 (5.26%)	4 (20.0%)	0.086
M2	40 (51.9%)	30 (52.6%)	10 (50.0%)	
M4	5 (6.49%)	5 (8.77%)	0 (0.00%)	
M5	24 (31.2%)	19 (33.3%)	5 (25.0%)	
M7	1 (1.30%)	0 (0.00%)	1 (5.00%)	
2017 ELN risk stratification				
favorable	19 (24.7%)	18 (31.6%)	1 (5.00%)	**0.008**
intermediate	25 (32.5%)	20 (35.1%)	5 (25.0%)	
adverse	33 (42.9%)	19 (33.3%)	14 (70.0%)	
Treatment response				
CR	59 (76.6%)	48 (84.2%)	11 (55.0%)	**0.001**
PR	5 (6.49%)	0 (0.00%)	5 (25.0%)	
NR	13 (16.9%)	9 (15.8%)	4 (20.0%)	

The bold values were statistically significant

**Table 2:** Genetic abnormality of AML patients respect to RCC1 expression

Genetic abnormality	total (n = 77)		low RCC1 expression (n = 57)		high RCC1 expression (n = 20)		*p*-value
	n	%	n	%	n	%	
RUNX1	7	9.1	5	8.8	2	10.0	1.00
TP53	7	9.1	3	5.3	4	20.0	0.07
ASXL1	11	14.3	7	12.3	4	20.0	0.46
GATA2	5	6.5	3	5.3	2	10.0	0.60
NPM1	11	14.3	10	17.5	1	5.0	0.27
CEBPA	6	7.8	5	8.8	1	5.0	1.00
FLT3	11	14.3	9	15.8	2	10.0	0.72
KMT2A rearrangement	6	7.8	2	3.5	4	20.0	**0.04**
t(8;21)/AML-ETO	7	9.1	6	10.5	1	5.0	0.67
CBFB-MYH11	6	7.8	6	10.5	0	0.0	0.33
DEK-NUP214	2	2.6	2	3.5	0	0.0	1.00
BCR-ABL1	1	1.3	1	1.8	0	0.0	1.00
−7/7q	8	10.4	5	8.8	3	15.0	0.42
+ 8	6	7.8	4	7.0	2	10.0	0.65
−5/5q	6	7.8	3	5.3	3	15.0	0.18
−17/17p	5	6.5	3	5.3	2	10.0	0.60
Normal karyotype	41	56.9	34	60.7	7	43.8	0.262
Complex karyotype	9	12.5	3	5.4	6	37.5	**0.003**

The bold values were statistically significant

### Targeting c-Myc/RCC1 signaling in the combination-induced anti-leukemia effect

**Fig. 9:**
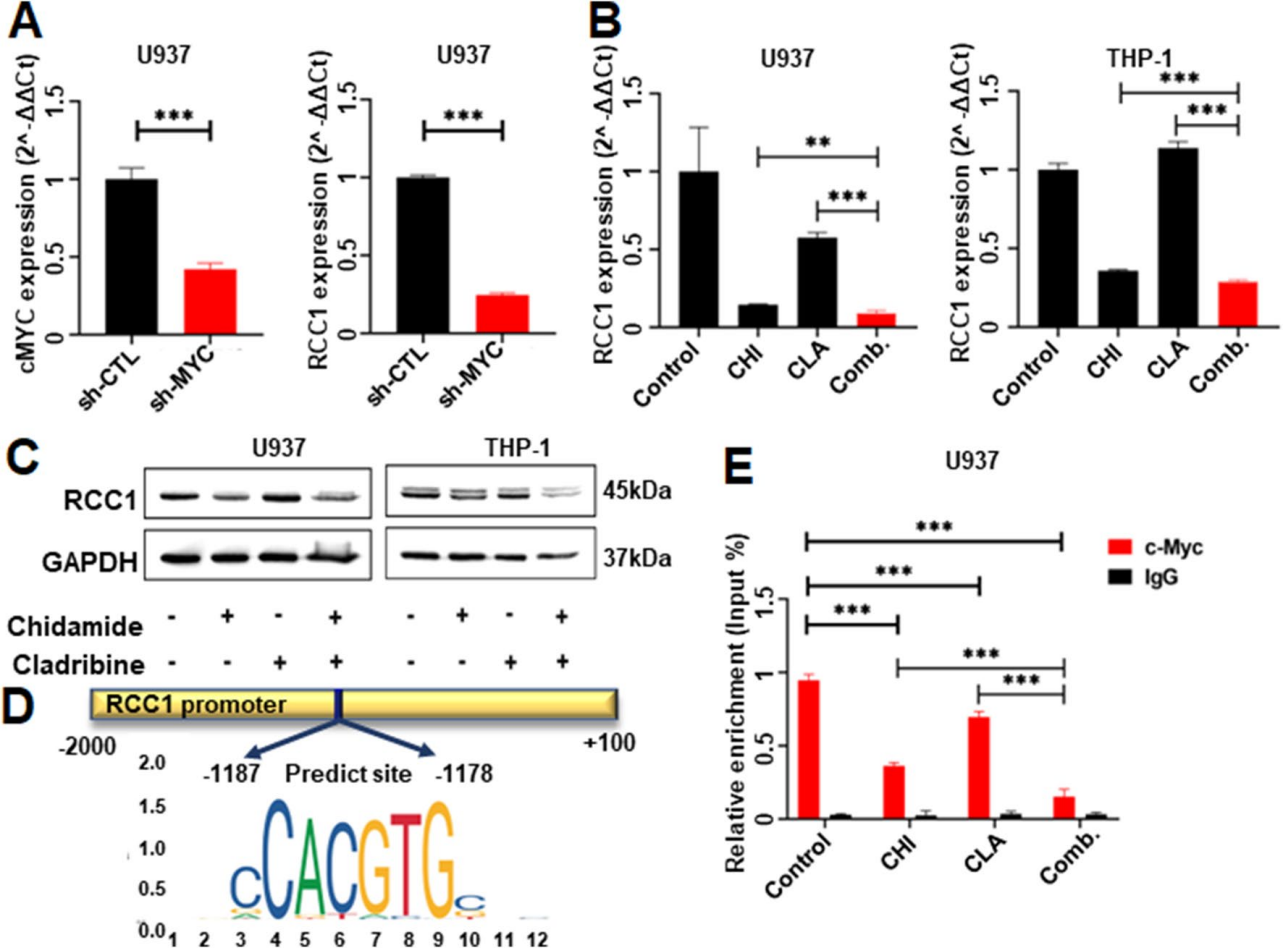
RCC1 as the target of c-Myc in AML. (**A**) The expression of c-MYC and RCC1 in vector control versus shMYC-U937 cells was detected by RT-qPCR. (**B**) The expression of RCC1 in U937 cells and THP-1 cells treated with Chidamide (CHI) alone, Cladribine (CLA) alone, and two drugs combination for 48 h was detected by RT-qPCR. (**C**) Western blot of RCC1 in U937 cells and THP-1 cells that were treated by CHI alone, CLA alone, and two drugs combination for 48 h. (**D**) The predicted binding sequence in the promoter region of RCC1 and the motif plot of the E-box element. (**E**) The relative amount of precipitated RCC1 promoter DNA of the treated cells was purified by c-Myc antibody and was detected by RT-qPCR. *p < 0.05, **p < 0.01, ***p < 0.001

We detected the c-MYC knockdown significantly induced the decrease of RCC1 mRNA level in the U937 cell line compared to shCTL (Fig. 9A). Also, RCC1 was significantly downregulated by the combination of CHI with CLA compared to DMSO control and also either single drug control in U937, THP-1, and MV4-11 (Fig. 9B, C and Additional file 15: Fig. S8A, B).

Moreover, we utilized the JASPAR online tool (https://jaspar.genereg.net/) to screen the potential binding motif of c-Myc in the promoter region of RCC1. Two putative binding sites (Fig. 9D) at − 1187 and − 1178 from the RCC1 transcription start site (TSS) were identified with a relative profile score threshold of 80%. qChIP was performed with the primers covering the sequence of putative binding sites. Results showed that c-Myc significantly binds to the promoter of RCC1 (Fig. 9E, left panel), and the combination significantly reduced c-Myc binding in the promoter region of RCC1 compared to the single drug control (Fig. 9E, right 3 panels). These data confirmed that RCC1 is the downstream effector of c-Myc in AML.

Taken together, these data indicated that the combination of CHI with CLA exerts its anti-leukemia effect by targeting c-Myc/RCC1 signaling in AML cells.

### The synergistic effect of CHI with CLA on cell growth arrest and apoptosis in AML patient samples

**Fig. 10:**
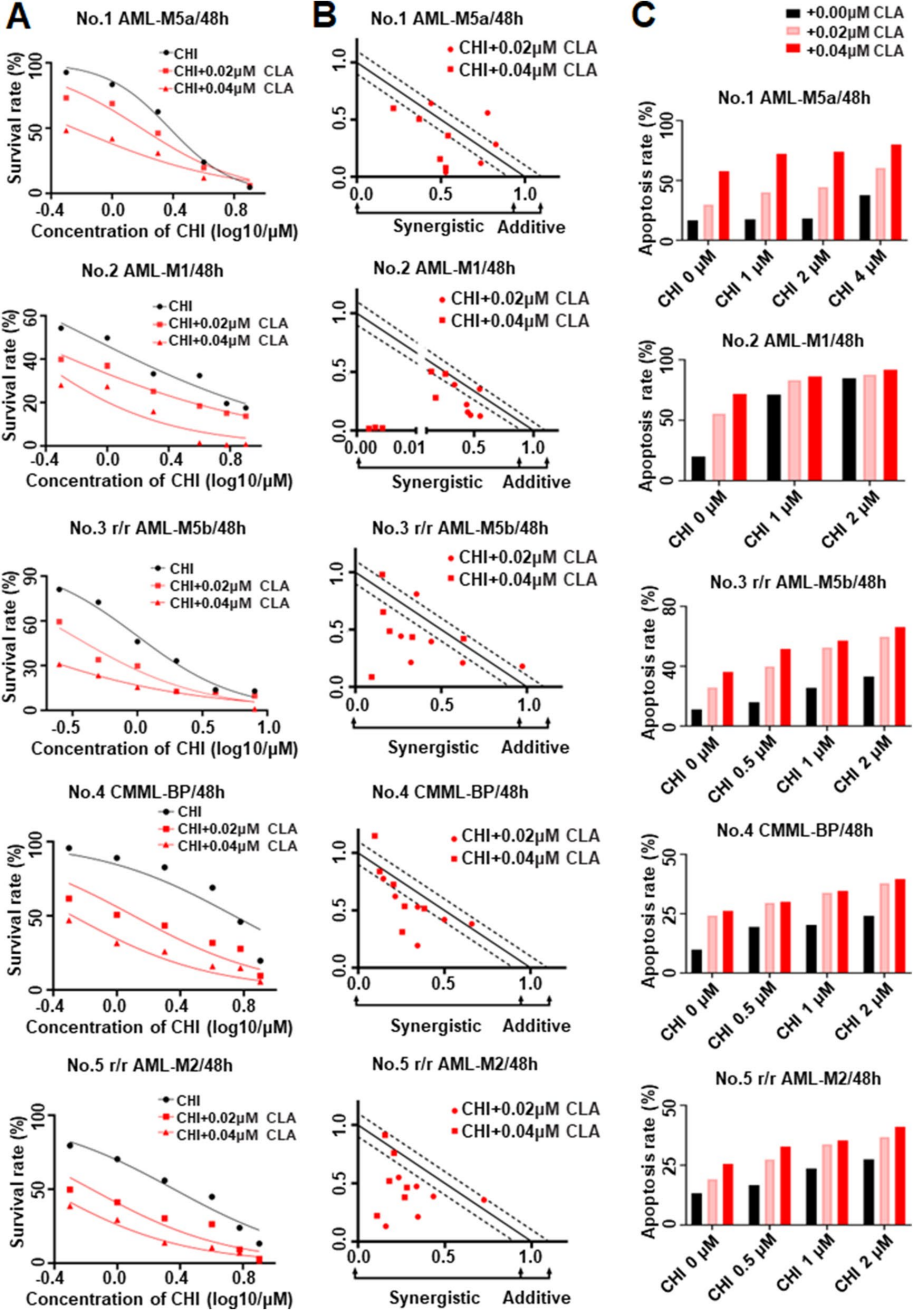
The synergistic effect of Chidamide (CHI) and Cladribine (CLA) in AML primary cells. The cell proliferation assays (**A**) and the normalized isobologram plots of the combination of CHI and CLA (**B**) in primary cells. The statistic histogram (**C**) of apoptosis effect for primary cells that were treated by gradient concentration of CHI alone, CLA alone, and two drugs combination for 48 h. *p < 0.05, **p < 0.01, ***p < 0.001

Finally, we examined the effect of the combination of CHI with CLA in primary cells from 5 AML patients (Additional file 1: Table S1). The IC50 of CLA on cell growth arrest was determined for 48 h (Additional file 15: Fig. S8C–G). Results showed that the combination of CHI with CLA in the dose of either IC50 or half IC50 significantly increased the cell growth arrest in the 5 patient samples compared to either single drug controls (Fig. 10A), and CompuSyn analysis showed the combination had the synergistic effect (Fig. 10B, Additional file 8: Table S6).

Similar results were observed for the effect of the combination on apoptosis for samples. Cells were treated with the combination of various doses of CHI (0.5 to 4μM) with different doses of CLA (0, 0.02, 0.04 μM), respectively. Results showed that the combination of CHI with CLA significantly increased apoptosis of primary cells in a dose-dependent manner (Fig. 10C, Additional file 16: Fig. S9A–E). The combination showed a much higher effect on apoptosis in the patient No.2 sample than that in the rest patients and also in a dose-dependent manner. These data indicated that the combination of CHI with CLA has a synergistic effect on cell growth arrest and apoptosis in primary cells, revealing the clinical application for AML therapy.

## Discussion

In this study, we first explored the effect of HDACi, CHI combined with CLA in AML. CHI plus low dose CLA could synergistically suppress the proliferation and introduce cell cycle arrest and apoptosis in AML cell lines and primary AML cells. Our results provide experimental evidence and potential molecular mechanisms to support the undergoing clinical trial (Clinical Trial ID: NCT05330364) on CHI combined with CLA to treat AML patients, especially who are intolerant to intensive chemotherapy.

We observed the effect of the combination on the primary cells from AML patients with different genetic abnormalities, MLL-AF6 rearrangement (patient I), AML1-ETO fusion gene co-existed with c-Kit mutation (patient II), R/R AML with FLT3-ITD mutation (Patient III), AML secondary to CMML (CMML-blast phase, CMML-BP) with complex karyotype and EZH2, RUNX1and SETBP1 mutations (Patient IV). These genetic defects are an indicator of poor outcomes [20–23]. Our data showed the synergistic cell proliferation arrest in the cells of the 2 de novo AML, 2 r/r AML and 1 CMML transformed AML by CHI combination with CLA. These data are not only consistent with the previous clinical trial reports and observations on CHI-based chemotherapy [21, 22, 24–26] but also revealed the potential efficacy of the combination on AML patients with poor outcomes with genetic defects.

C-Myc is the key target of HDACi in cancer [27]. Since we observed CHI alone could significantly down-regulate c-Myc in AML cell lines, we speculated there is an HDACs/c-Myc complex. We first observed the interaction of HDAC2 and c-Myc in AML cells and RCC1 is the downstream effector of c-Myc in AML cells as reported in solid tumors [28]. RCC1 is reported to regulate the cell cycle by targeting the cycle-related kinases CDK2/Cyclin E2 and their inhibitor, p21^CIP1^ [29], and to promote apoptosis by activating caspase-9 [30]. C-Myc, as well as RCC1, were further down-regulated by CHI plus CLA. We also observed the elevated RCC1 expression in AML patients and the high RCC1 expression associated with adverse outcomes as in solid cancers [31]. Also, obvious c-Myc binding peaks are observed in the promoter region of RCC1 by ChIP-seq (data not shown), c-Myc significantly binds to the promoter of RCC1 in U937 cells, and the combination significantly reduced c-Myc binding in the promoter region of RCC1 compared to the single drug control, and c-Myc knockdown induces RCC1 down-regulation in AML cells, indicating CHI plus CLA downregulated RCC1 expression in a c-MYC-dependent manner. Our results indicated a new oncogenic signaling pathway, i.e. HDAC2/c-Myc/RCC1 signaling in AML cells. The combination of CHI and CLA suppressed this oncogenic signaling to induce the G0/G1 phase arrest and apoptosis, which caused the anti-leukemia effect (Additional file 17: Fig. S10).

## Conclusions

Our study showed synergistic effects of CHI combined with CLA on cell proliferation arrest, apoptosis, and cell cycle arrest by targeting HDAC2/c-Myc/RCC1 signaling in AML cells. Our results provide the pre-clinical evidence for the ongoing clinical trial (Clinical Trial ID: NCT05330364) concerning the combined therapy of CHI with CLA in AML patients.

## Supplementary Information

**Additional file 1: Table S1.** Clinical features of 5 AML patients.

**Additional file 2: Table S2.** The ChIP-seq scores of c-Myc binding targets in hematopoietic stem and progenitor cells.

**Additional file 3: Table S3.** The shRNA oligos and primers.

**Additional file 4: Table S4.** The primary antibodies.

**Additional file 5: Table S5.** The half maximal inhibitory concentration of Chidamide in AML cell lines.

**Additional file 6: Figure S1.** The inhibition effects of Chidamide (CHI) alone in MV4-11 cells. (A) The cell proliferation assays in MV4-11 cells were treated by CHI alone with gradient concentration for 24, 48, and 72 h. The statistic histogram (B) and flow cytometry histogram (C) of cell cycle effect, The statistic histogram (D) and flow cytometry scatter plot (E) of apoptosis effect in MV4-11 cells that were treated by CHI alone with gradient concentration for 48 h. * *p* < 0.05, ** *p* < 0.01, *** *p* < 0.001.

**Additional file 7: Figure S2.** The inhibition effects of Chidamide (CHI) alone, Cladribine (CLA) alone, or in combination on PBMC from healthy donors and AML cell lines. The cell proliferation assays in PBMC were treated with low gradient concentration (A) or high gradient concentration (C) CHI and combined with 0.02 μM CLA for 48 h. The cell proliferation assays in PBMC were treated with low gradient concentration (B) or high gradient concentration (D) CLA. The inhibition effects of CLA alone in (E) U937, (F) THP-1, and (G) MV4-11 cells for 48 h. (H) The cell proliferation assays in MV4-11 cells were treated by CHI alone with gradient concentration or CHI combined with 0.01 μM or 0.02 μM CLA for 48 h. (I) The normalized isobologram plot of the combination of CHI and CLA in MV4-11 cells. * *p* < 0.05, ** *p* < 0.01, *** *p* < 0.001.

**Additional file 8: Table S6.** The combination index value of Chidamide plus Cladribine in AML cells.

**Additional file 9: Figure S3.** The synergistic cell cycle effect of Chidamide (CHI) and Cladribine (CLA) in MV4-11 cells. The flow cytometry histogram (A) and statistic histogram (B) of cell cycle effect; (C) western blot of cyclin E2, CDK2, and p21 ^Waf1/Cip1^ in MV4-11 cells that were treated by CHI alone, CLA alone, and two drugs combination for 48 h. * *p* < 0.05, ** *p* < 0.01, *** *p* < 0.001.

**Additional file 10: Figure S4.** The synergistic apoptosis effect of Chidamide (CHI) and Cladribine (CLA) in MV4-11 cells. The flow cytometry scatters plot (A) and statistic histogram (B) of apoptosis effect; (C) western blot of cleaved caspase-9, cleaved caspase-3, cleaved PARP in MV4-11 cells that were treated by CHI alone, CLA alone, and two drugs combination for 48 h. * *p* < 0.05, ** *p* < 0.01, *** *p* < 0.001.

**Additional file 11: Figure S5.** Enrichment analysis referred to (A) The Hallmark Gene Sets, (B) Canonical Pathways, (C) Chemical and Genetic Perturbations, and (D) Oncogenic Signatures datasets. (E) The expression of c-MYC in MV4-11 cells treated with Chidamide (CHI) alone, Cladribine (CLA) alone, and two drugs combination for 48 h was detected by RT-qPCR. (F) Western blot of c-Myc in MV4-11 cells that were treated by CHI alone, CLA alone, and two drugs combination for 48 h. (G) The expression of c-Myc in AML cell lines. * *p* < 0.05, ** *p* < 0.01, *** *p* < 0.001.

**Additional file 12: Figure S6.** (A) Co-Immunoprecipitation analysis was for c-Myc, HDAC1, and HDAC2 in MV4-11 cells. (B) The expression of HDAC2 in AML cell lines. The different expression levels of HDACs in AML patients versus normal donors in the GSE13159 (C) and GSE114868 (D) datasets. The survival curve of AML patients was grouped into high- or low- HDAC2 expression and was analyzed in GSE12417-GPL570 (E), and GSE37642- GPL570 (F) datasets. * *p* < 0.05, ** *p* < 0.01, *** *p* < 0.001.

**Additional file 13: Table S7.** Clinical characteristics and genetic abnormalities of AML patients respect to HDAC2 expression.

**Additional file 14: Figure S7.** The significance of the RCC1 in AML. (A) the Venn plot of the co-expression differential expression genes regulated by two drugs and the potential c-MYC target genes. RCC1 was positively regulated with c-MYC in AML patients analyzed in the TCGA (B), GSE995 (C), and GSE5122 datasets (D). The elevated expression levels of RCC1 in AML patients versus normal donors in the GSE13159 (E) and GSE114868 (F) datasets. The survival curve of AML patients was grouped into high- or low- RCC1 expression and was analyzed in TCGA (G), GSE12417-GPL570 (H), and GSE37642- GPL570 (I) datasets. (J) The potential transcription factor for RCC1 was analyzed by Cistrome online tool (http://cistrome.org/db/). * *p* < 0.05, ** *p* < 0.01, *** *p* < 0.001.

**Additional file 15: Figure S8.** (A) The expression of RCC1 in MV4-11 cells treated with Chidamide (CHI) alone, Cladribine (CLA) alone, and two drugs combination for 48 h was detected by RT-qPCR. (B) Western blot of RCC1 in MV4-11 cells that were treated by CHI alone, CLA alone, and two drugs combination for 48 h. (C-G) The inhibition effects of CLA alone in AML primary cells. * *p* < 0.05, ** *p* < 0.01, *** *p* < 0.001.

**Additional file 16: Figure S9.** The scatters plots of apoptosis effect for AML primary cells that were treated by gradient concentration of Chidamide alone, Cladribine alone, and two drugs combination for 48 h.

**Additional file 17: Figure S10.** The mechanism of the synergistic effect of Chidamide and Cladribine in AML.

## Data Availability

The data generated during the current study are available from the corresponding author upon reasonable request. The datasets analyzed during the current study are available in Gene Expression Omnibus (https://www.ncbi.nlm.nih.gov/geo/) database or The Cancer Genome Atlas (TCGA, https://portal.gdc.cancer.gov/).

## Abbreviations

AML: Acute myeloid leukemia
CR: Complete remission
HDACi: Histone deacetylase inhibitor
CHI: Chidamide
CLA: Cladribine
RT-qPCR: Real-time quantitative polymerase chain reaction
DMSO: Dimethyl sulfoxide
RNA-seq: RNA-sequence
TCGA: The Cancer Genome Atlas
GEO: Gene Expression Omnibus
CCK-8: Cell counting kit-8
IC50: The 50% inhibitory concentration
CI: Combination index
PI: Propidium iodide
IP: Immunoprecipitation
WB: Western blot
ChIP: Chromatin immunoprecipitation
DEG: Differential expression gene
shCTL: Scramble shRNA control
CMML-BP: Chronic myelomonocytic leukemia blast phase
