# Supplementary figures and images for: Synergistic effect of HDAC inhibitor Chidamide with Cladribine on cell cycle arrest and apoptosis by targeting HDAC2/c-Myc/RCC1 axis in acute myeloid leukemia
Source: Exp Hematol Oncol. 2023 Feb 27;12:23. doi: 10.1186/s40164-023-00383-5 (PMC9972767; doi:10.1186/s40164-023-00383-5)

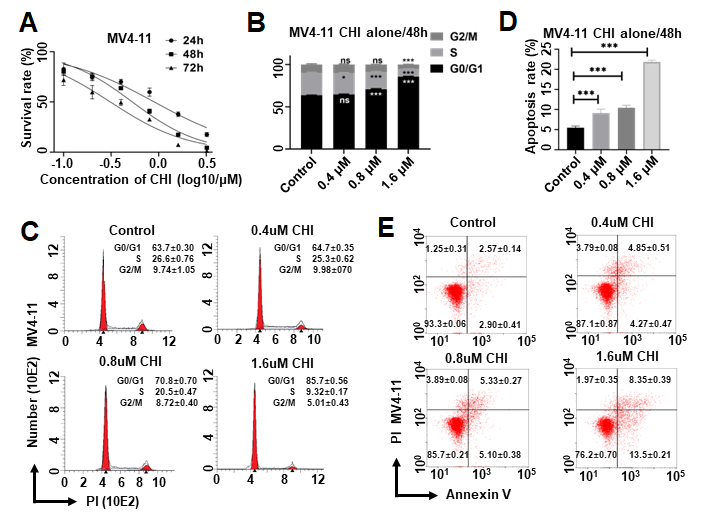

Supplement: Supplementary file 6 — Additional file 6: Figure S1. The inhibition effects of Chidamide (CHI) alone in MV4-11 cells. (A) The cell proliferation assays in MV4-11 cells were treated by CHI alone with gradient concentration for 24, 48, and 72 h. The statistic histogram (B) and flow cytometry histogram (C) of cell cycle effect, The statistic histogram (D) and flow cytometry scatter plot (E) of apoptosis effect in MV4-11 cells that were treated by CHI alone with gradient concentration for 48 h. * p < 0.05, ** p < 0.01, *** p < 0.001. [file 40164_2023_383_MOESM6_ESM.tif]

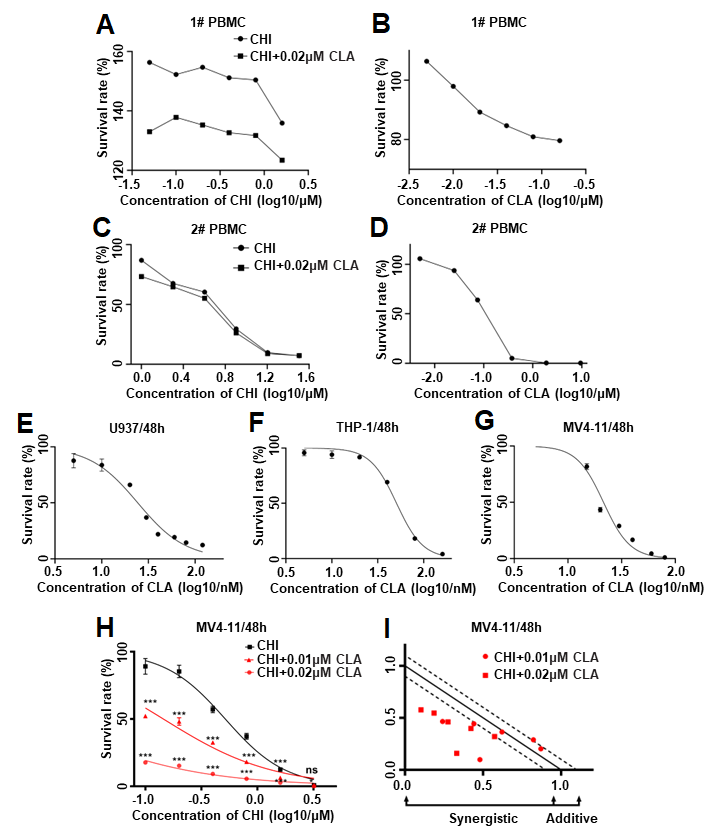

Supplement: Supplementary file 7 — Additional file 7: Figure S2. The inhibition effects of Chidamide (CHI) alone, Cladribine (CLA) alone, or in combination on PBMC from healthy donors and AML cell lines. The cell proliferation assays in PBMC were treated with low gradient concentration (A) or high gradient concentration (C) CHI and combined with 0.02 μM CLA for 48 h. The cell proliferation assays in PBMC were treated with low gradient concentration (B) or high gradient concentration (D) CLA. The inhibition effects of CLA alone in (E) U937, (F) THP-1, and (G) MV4-11 cells for 48 h. (H) The cell proliferation assays in MV4-11 cells were treated by CHI alone with gradient concentration or CHI combined with 0.01 μM or 0.02 μM CLA for 48 h. (I) The normalized isobologram plot of the combination of CHI and CLA in MV4-11 cells. * p < 0.05, ** p < 0.01, *** p < 0.001. [file 40164_2023_383_MOESM7_ESM.tif]

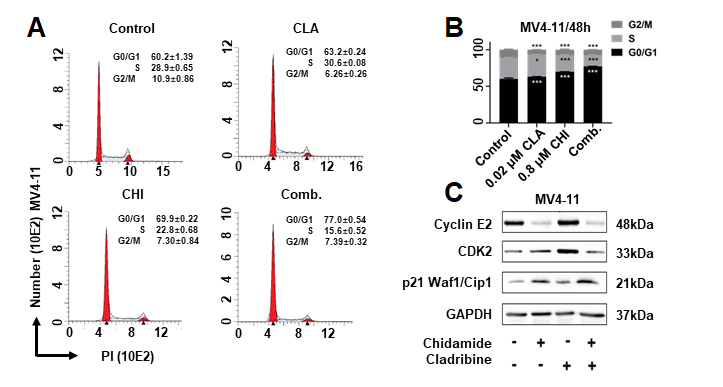

Supplement: Supplementary file 9 — Additional file 9: Figure S3. The synergistic cell cycle effect of Chidamide (CHI) and Cladribine (CLA) in MV4-11 cells. The flow cytometry histogram (A) and statistic histogram (B) of cell cycle effect; (C) western blot of cyclin E2, CDK2, and p21 Waf1/Cip1 in MV4-11 cells that were treated by CHI alone, CLA alone, and two drugs combination for 48 h. * p < 0.05, ** p < 0.01, *** p < 0.001. [file 40164_2023_383_MOESM9_ESM.tif]

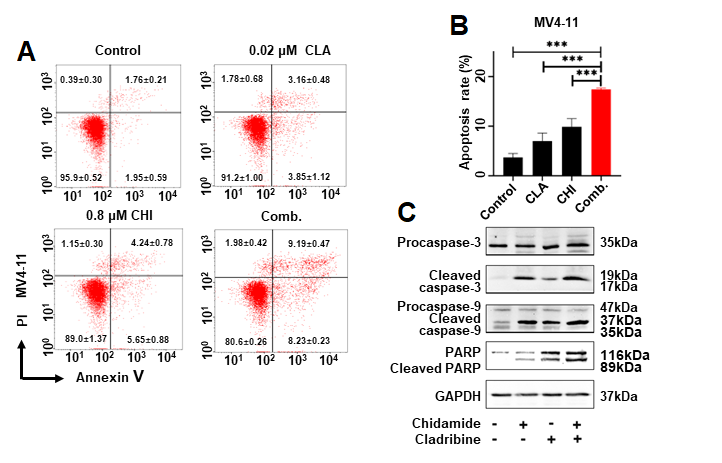

Supplement: Supplementary file 10 — Additional file 10: Figure S4. The synergistic apoptosis effect of Chidamide (CHI) and Cladribine (CLA) in MV4-11 cells. The flow cytometry scatters plot (A) and statistic histogram (B) of apoptosis effect; (C) western blot of cleaved caspase-9, cleaved caspase-3, cleaved PARP in MV4-11 cells that were treated by CHI alone, CLA alone, and two drugs combination for 48 h. * p < 0.05, ** p < 0.01, *** p < 0.001. [file 40164_2023_383_MOESM10_ESM.tif]

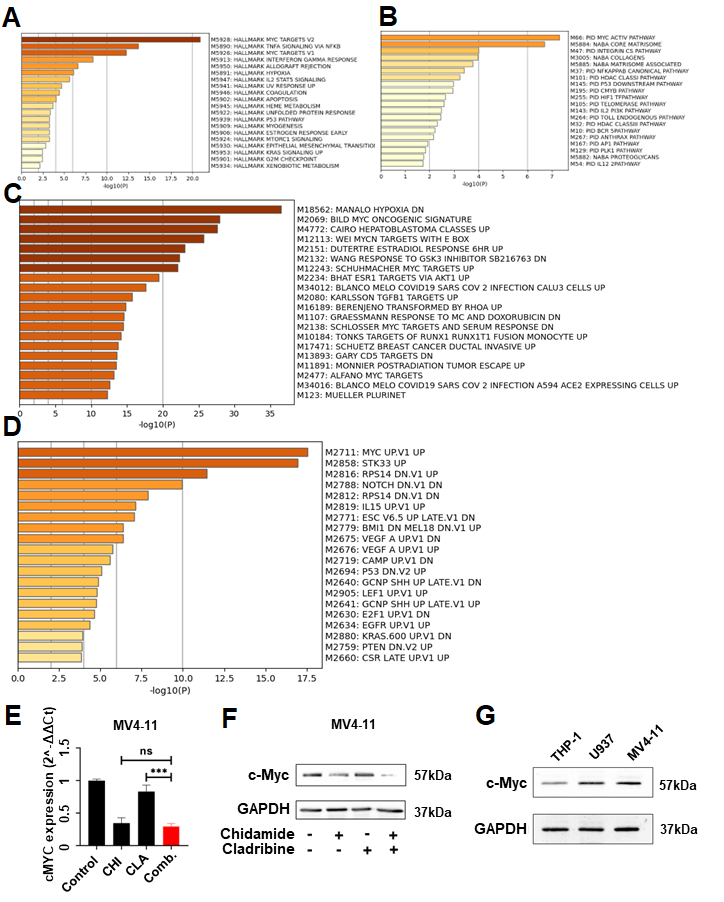

Supplement: Supplementary file 11 — Additional file 11: Figure S5. Enrichment analysis referred to (A) The Hallmark Gene Sets, (B) Canonical Pathways, (C) Chemical and Genetic Perturbations, and (D) Oncogenic Signatures datasets. (E) The expression of c-MYC in MV4-11 cells treated with Chidamide (CHI) alone, Cladribine (CLA) alone, and two drugs combination for 48 h was detected by RT-qPCR. (F) Western blot of c-Myc in MV4-11 cells that were treated by CHI alone, CLA alone, and two drugs combination for 48 h. (G) The expression of c-Myc in AML cell lines. * p < 0.05, ** p < 0.01, *** p < 0.001. [file 40164_2023_383_MOESM11_ESM.tif]

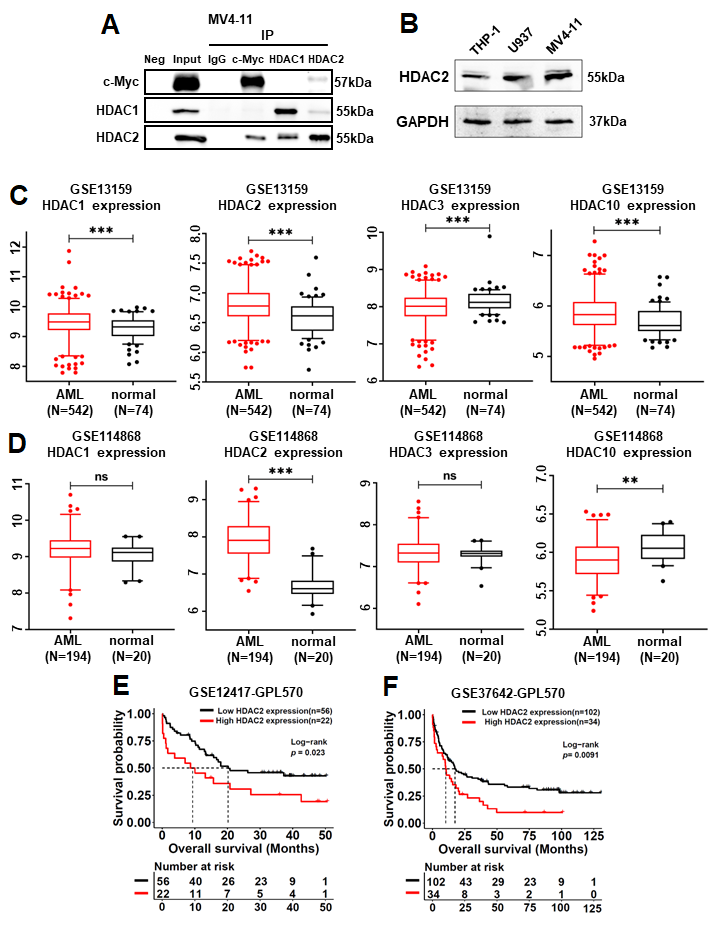

Supplement: Supplementary file 12 — Additional file 12: Figure S6. (A) Co-Immunoprecipitation analysis was for c-Myc, HDAC1, and HDAC2 in MV4-11 cells. (B) The expression of HDAC2 in AML cell lines. The different expression levels of HDACs in AML patients versus normal donors in the GSE13159 (C) and GSE114868 (D) datasets. The survival curve of AML patients was grouped into high- or low- HDAC2 expression and was analyzed in GSE12417-GPL570 (E), and GSE37642- GPL570 (F) datasets. * p < 0.05, ** p < 0.01, *** p < 0.001. [file 40164_2023_383_MOESM12_ESM.tif]

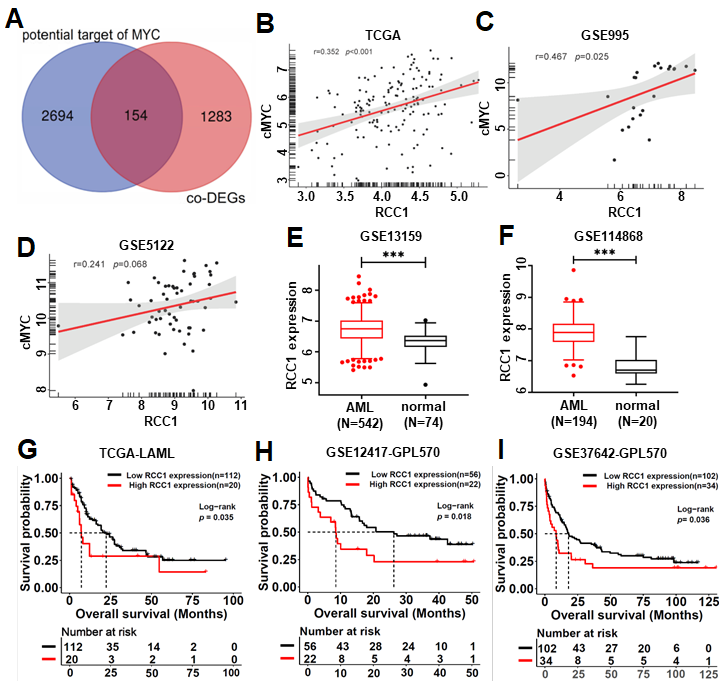

Supplement: Supplementary file 14 — Additional file 14: Figure S7. The significance of the RCC1 in AML. (A) the Venn plot of the co-expression differential expression genes regulated by two drugs and the potential c-MYC target genes. RCC1 was positively regulated with c-MYC in AML patients analyzed in the TCGA (B), GSE995 (C), and GSE5122 datasets (D). The elevated expression levels of RCC1 in AML patients versus normal donors in the GSE13159 (E) and GSE114868 (F) datasets. The survival curve of AML patients was grouped into high- or low- RCC1 expression and was analyzed in TCGA (G), GSE12417-GPL570 (H), and GSE37642- GPL570 (I) datasets. (J) The potential transcription factor for RCC1 was analyzed by Cistrome online tool (http://cistrome.org/db/). * p < 0.05, ** p < 0.01, *** p < 0.001. [file 40164_2023_383_MOESM14_ESM.tif]

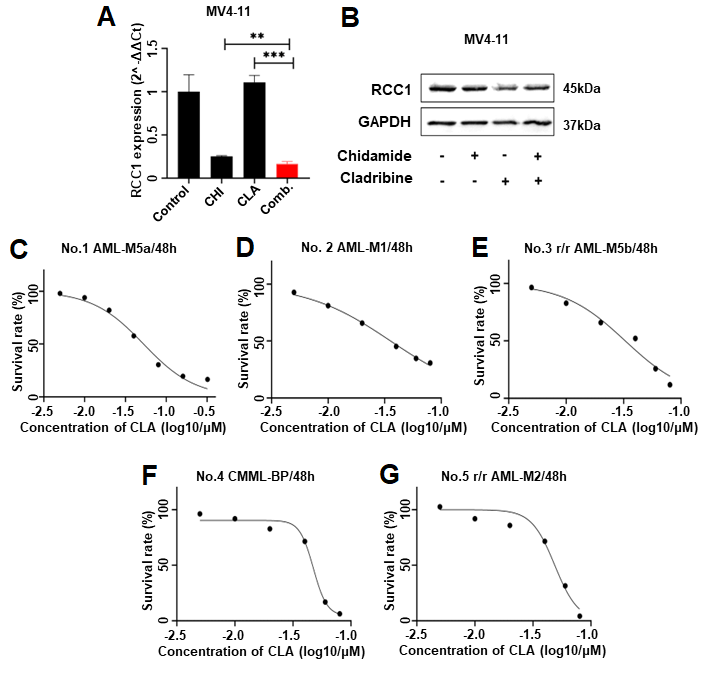

Supplement: Supplementary file 15 — Additional file 15: Figure S8. (A) The expression of RCC1 in MV4-11 cells treated with Chidamide (CHI) alone, Cladribine (CLA) alone, and two drugs combination for 48 h was detected by RT-qPCR. (B) Western blot of RCC1 in MV4-11 cells that were treated by CHI alone, CLA alone, and two drugs combination for 48 h. (C-G) The inhibition effects of CLA alone in AML primary cells. * p < 0.05, ** p < 0.01, *** p < 0.001. [file 40164_2023_383_MOESM15_ESM.tif]

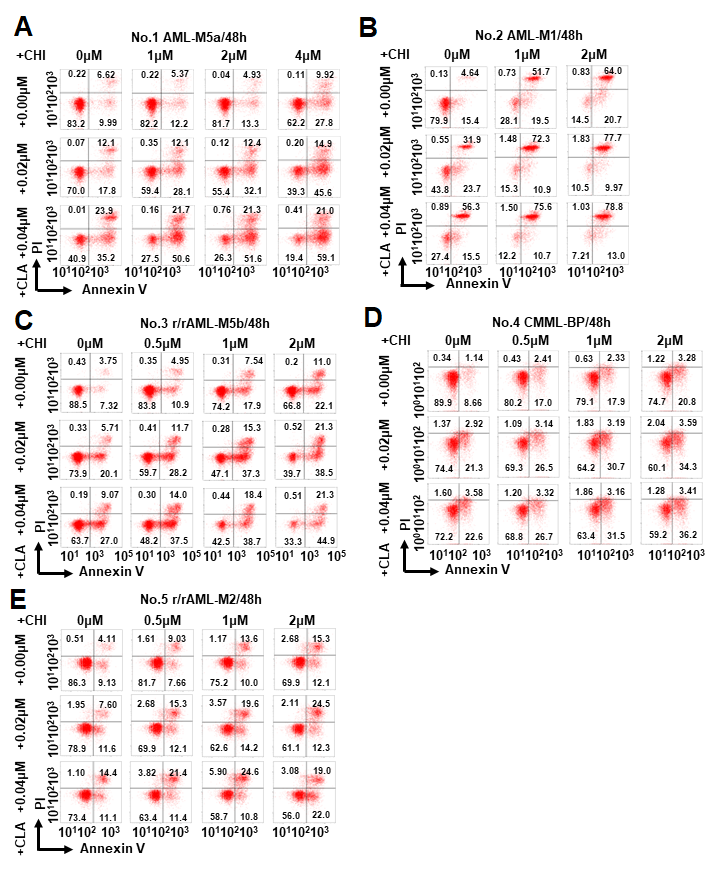

Supplement: Supplementary file 16 — Additional file 16: Figure S9. The scatters plots of apoptosis effect for AML primary cells that were treated by gradient concentration of Chidamide alone, Cladribine alone, and two drugs combination for 48 h. [file 40164_2023_383_MOESM16_ESM.tif]

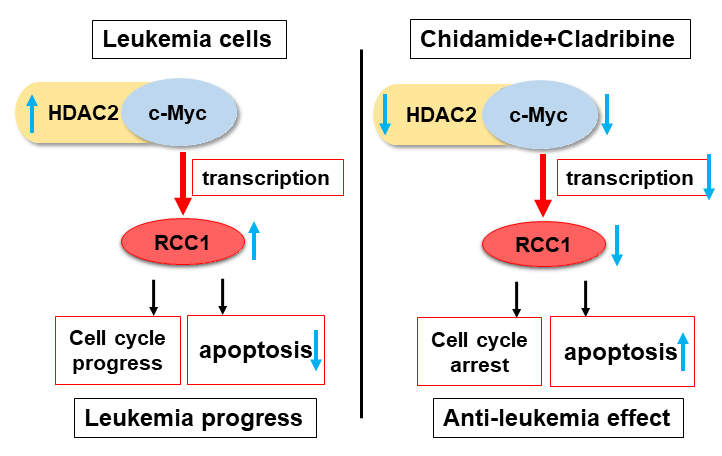

Supplement: Supplementary file 17 — Additional file 17: Figure S10. The mechanism of the synergistic effect of Chidamide and Cladribine in AML. [file 40164_2023_383_MOESM17_ESM.tif]
