# Supplementary material for: Synergistic effect of HDAC inhibitor Chidamide with Cladribine on cell cycle arrest and apoptosis by targeting HDAC2/c-Myc/RCC1 axis in acute myeloid leukemia
Source: Exp Hematol Oncol. 2023 Feb 27;12:23. doi: 10.1186/s40164-023-00383-5 (PMC9972767; doi:10.1186/s40164-023-00383-5)
Supplement: Supplementary file 13 — Additional file 13: Table S7. Clinical characteristics and genetic abnormalities of AML patients respect to HDAC2 expression. [file 40164_2023_383_MOESM13_ESM.docx]

| **Characteristics/** **genetic abnormalities** | **total (n=77)** | **low HDAC2 expression (n=57)** | **high HDAC2 expression (n=20)** | ***p*-value** |
| --- | --- | --- | --- | --- |
|  | **n (%) or median (range)** | **n (%) or median (range)** | **n (%) or median (range)** |  |
| **Gender (Male)** | 40 (51.9%) | 31 (54.4%) | 9 (45.0%) | 0.604 |
| **Age (years)** | 60 (18-86) | 64 (18-86) | 57 (21-78) | 0.317 |
| **WBC (x10^9/L)** | 7.71 (0.4-123.6) | 7.79 (0.4-123.6) | 5.59 (1.05-101.82) | 0.736 |
| **Hb (g/L)** | 75 (36-152) | 77 (36-152) | 69 (49-109) | 0.296 |
| **Plt (x10^9/L)** | 53 (3-259) | 62 (9-184) | 36.5 (3-259) | 0.079 |
| **BM blast (%)** | 46 (6.05-96) | 52 (6.05-96) | 42 (6.4-95.2) | 0.095 |
| **FAB** |  |  |  |  |
| **M1** | 7 (9.1%) | 6 (10.5%) | 1 (5%) | 0.476 |
| **M2** | 40 (51.9%) | 30 (52.6%) | 10 (50.0%) |  |
| **M4** | 5 (6.5%) | 5 (8.8%) | 0 (0.00%) |  |
| **M5** | 24 (31.2%) | 15 (26.3%) | 9 (45.0%) |  |
| **M7** | 1 (1.3%) | 1 (1.8%) | 0 (0.00%) |  |
| **2017 ELN risk stratification** |  |  |  |  |
| **favorable** | 19 (24.7%) | 17 (29.8%) | 2 (10.00%) | 0.203 |
| **intermediate** | 25 (32.5%) | 18 (31.6%) | 7 (35.0%) |  |
| **adverse** | 33 (42.9%) | 22 (38.6%) | 11 (55.0%) |  |
| **Treatment response** |  |  |  |  |
| **CR** | 59 (76.6%) | 47 (82.5%) | 12 (60.0%) | 0.094 |
| **PR** | 5 (6.50%) | 3 (5.3%) | 2 (10.0%) |  |
| **NR** | 13 (16.9%) | 7 (12.3%) | 6 (30.0%) |  |
| **RUNX1** | 7(9.1%) | 4(7.0%) | 3(15.0%) | 0.367 |
| **TP53** | 7(9.1%) | 3(5.3%) | 4(20.0%) | 0.070 |
| **ASXL1** | 11(14.3%) | 6(10.5%) | 5(25.0%) | 0.141 |
| **GATA2** | 5(6.5%) | 2(3.5%) | 3(15.0%) | 0.107 |
| **NPM1** | 11(14.3%) | 9(15.8%) | 2(10.0%) | 0.718 |
| **CEBPA** | 6(7.8%) | 4(7.0%) | 2(10.0%) | 0.647 |
| **FLT3** | 11(14.3%) | 11(19.3%) | 0(0.0%) | 0.057 |
| **KMT2A rearrangement** | 6(7.8%) | 4(7.0%) | 2(10.0%) | 0.647 |
| **t(8;21)/AML-ETO** | 7(9.1%) | 6(10.5%) | 1(5.0%) | 0.669 |
| **CBFB-MYH11** | 6(7.8%) | 5(8.8%) | 1(5.0%) | 1.000 |
| **DEK-NUP214** | 2(2.6%) | 2(3.5%) | 0(0.0%) | 1.000 |
| **BCR-ABL1** | 1(1.3%) | 0(0.0%) | 1(5.0%) | 0.260 |
| **-7/7q** | 8(10.4%) | 5(8.8%) | 3(15.0%) | 0.421 |
| **+8** | 6(7.8%) | 5(8.8%) | 1(5.0%) | 1.000 |
| **-5/5q** | 6(7.8%) | 2(3.5%) | 4(20.0%) | **0.037** |
| **-17/17p** | 5(6.5%) | 4(7.0%) | 1(5.0%) | 1.000 |
| **normal karyotype** | 41(56.9%) | 31(58.5%) | 10(52.6%) | 0.788 |
| **complex karyotype** | 9(12.5%) | 5(9.4%) | 4(21.1%) | 0.231 |

Table S7. Clinical characteristics and genetic abnormalities of AML patients respect to HDAC2 expression
